# Highly branched and loop-rich gels via formation of metal–organic cages linked by polymers

**DOI:** 10.1038/nchem.2390

**Published:** 2015-11-16

**Authors:** Aleksandr V. Zhukhovitskiy, Mingjiang Zhong, Eric G. Keeler, Vladimir K. Michaelis, Jessie E. P. Sun, Michael J. A. Hore, Darrin J. Pochan, Robert G. Griffin, Adam P. Willard, Jeremiah A. Johnson

**Affiliations:** 1 Department of Chemistry, Massachusetts Institute of Technology, 77 Massachusetts Avenue, Cambridge, Massachusetts 02139, USA; 2 Francis Bitter Magnet Laboratory, Massachusetts Institute of Technology, 77 Massachusetts Avenue, Cambridge, Massachusetts 02139, USA; 3 Department of Materials Science and Engineering, University of Delaware, 201 DuPont Hall, Newark, Delaware 19716, USA; 4 Department of Macromolecular Science and Engineering, Case Western Reserve University, Cleveland, Ohio 44106, USA

## Abstract

Gels formed via metal–ligand coordination typically have very low branch functionality, *f*, as they consist of ∼2–3 polymer chains linked to single metal ions that serve as junctions. Thus, these materials are very soft and unable to withstand network defects such as dangling ends and loops. We report here a new class of gels assembled from polymeric ligands and metal-organic cages (MOCs) as junctions. The resulting ‘polyMOC’ gels are precisely tunable and may feature increased branch functionality. We show two examples of such polyMOCs: a gel with a low *f* based on a M_2_L_4_ paddlewheel cluster junction and a compositionally isomeric one of higher *f* based on a M_12_L_24_ cage. The latter features large shear moduli, but also a very large number of elastically inactive loop defects that we subsequently exchanged for functional ligands, with no impact on the gel's shear modulus. Such a ligand substitution is not possible in gels of low *f*, including the M_2_L_4_-based polyMOC.

Coordination chemistry typically features bonds that are intermediate in bond energy between covalent bond energy and other non-covalent interaction energies (for example, van der Waals interactions and hydrogen bonding)^1^. Such bonds have been used extensively for the formation of supramolecular polymer networks/gels^2–17^, metal–organic cages (MOCs)^18–25^ and metal–organic frameworks (MOFs)^26–28^; these important classes of materials feature an array of exciting, complementary properties. Materials that incorporate structural features that blend these classes of materials not only capitalize on their individual positive qualities, but also, by way of synergy, potentially exhibit unprecedented and valuable properties^29–31^.

A key component of any material structure is the network branch functionality, *f*, which is the average number of bridges that connect network junctions. In gels prepared from flexible polymers, an increase in *f* leads to a direct increase in the elastic modulus^32^. Existing supramolecular metallogels (for example, based on the coordination of Fe^3+^ and catechol derivatives and structural analogues^11,15^) have single metal atoms at their junctions (Fig. 1a, left), and these metals can typically only bind to 2–3 ligands. Thus, the ability to tune *f* in these systems is limited. In sharp contrast, MOCs and the junctions of MOFs typically comprise metal– ligand clusters with M*_i_*L*_j_* junction stoichiometry in which *j* ≥ *i* > 1. This augmented stoichiometry and increased junction functionality translates into unique cavity structures, but has little impact on viscoelasticity because MOCs and MOFs are generally rigid materials^33^.

With these considerations in mind and inspired by MOC synthesis^18–23^, we wondered if it would be possible to use a multi-metal–ligand supramolecular assembly to drive gelation and yield gels that consist of MOCs linked together by polymers— referred to as polyMOCs. These gels would feature tunable nanoscale junction structures and an enhanced *f* (Fig. 1a, right). Such an approach would be distinct from traditional supramolecular polymerizations^34,35^ that generate point-like junctions (Fig. 1a, left), or the pre-assembly of stable M*_i_*L*_j_* cages followed by aggregation or weak supramolecular crosslinking of these spectator cages^36–42^. To our knowledge, the concept of gelation driven by multicomponent M*_i_*L*_j_* assembly has been considered in only two reports, both of which focus on materials with a low *f*. First, we described the synthesis of hydrogels with targeted M_4_L_4_ square junctions via the assembly of Fe^2+^ or Ni^2+^ ions with bispyridyl tetrazine ligands bound to the ends of polyethylene glycol (PEG) chains^43^. Although gelation in this system was only possible through a multi-metal–ligand assembly, we could not characterize conclusively the putative M_4_L_4_ clusters, and we proposed that a mixture of clusters of different size was probably present. Nitschke and co-workers later reported hydrogels with targeted M_4_L_4_ pyramidal junctions prepared from the assembly of Fe^2+^ ions and 4,4′-diaminobiphenyl-2,2′-disulfonic acid ligands bound to the ends of PEG^44^. Small-molecule analogues of these ligands did form the target cages in solution, but the cages were not characterized directly in the analogous gels; uptake and release of small molecules from the materials suggested the presence of cavities^45^ with distinct environments. Although these examples are encouraging, tuning and enhancing *f* to enable unprecedented mechanical behaviours in the polyMOC context has not yet been demonstrated.

We began this study with two hypotheses. First, we reasoned that the thermal annealing of a mixture of Pd^2+^ ions and PEG terminated with *para*-bispyridyl ligands designed to form M_12_L_24_ cages^46,47^ (polymer ligand **PL1** (Fig. 1b)) or *meta*-bispyridyl ligands designed to form M_2_L_4_ paddlewheels^48,49^ (**PL2** (Fig. 1c)) would generate polyMOC gels, **gel-1** and **gel-2**, respectively, with junction structures similar to those of the target assemblies. Second, we proposed that the difference in average junction size, and the corresponding number of polymer chains connected to each cluster, would translate directly into changes in *f* and defects (for example, primary loops in which both ligand ends of a single polymer chain are attached to the same junction (red chains, Fig. 1a)) that would lead to unique mechanical properties. Here we use ^1^H magic-angle spinning (MAS) nuclear magnetic resonance (NMR) spectroscopy, small-angle neutron scattering (SANS), molecular dynamics simulations and oscillatory rheometry to test these hypotheses. Our results provide direct evidence for cage assembly in polyMOCs and show that gelation driven by a metal–ligand multi-component assembly programmed by small changes in ligand structure offers a powerful means to tune the network structure and mechanical properties. We demonstrate that the structure of **gel-1** (Fig. 1a, far right), which features a high *f* and also a large number of loop defects, can be leveraged to replace defects selectively with functional free ligands. Thus, materials with modified junctions can be produced with little impact on the shear modulus, which is not possible in polyMOC **gel-2** with a low *f*.

## Results

### Solution assembly of free ligands

We first confirmed that bispyridine ligands similar to those on the ends of **PL1** and **PL2** but not bound to a polymer (‘free ligands’ **L1** and **L2** (Fig. 2)) form the expected M_12_L_24_ and M_2_L_4_ assemblies, respectively, in the presence of Pd^2+^. Information from studies with these free ligands and their resulting MOCs is used below to validate the structure of polyMOCs. Exposure of **L1** to Pd(NO_3_)_2_·2H_2_O in dimethylsulfoxide (DMSO)-*d*_6_ (0.100 M) at room temperature (r.t.) provided a heterogeneous mixture with highly broadened ^1^H NMR resonances shifted to a higher frequency compared with free **L1** (Fig. 2a). This mixture transformed into a clear light-yellow solution on heating for eight hours at 70 °C. The ^1^H NMR spectrum of this solution contained one set of ligand-based resonances consistent with a highly symmetric nanoscopic assembly (Fig. 2a and Supplementary Fig. 10). The ^1^H NMR resonances in the aromatic region were shifted to a higher frequency compared with those of **L1**, and the corresponding chemical shifts were virtually identical to those reported by Fujita and co-workers for a similar system^46^.

On mixing **L2** with Pd(NO_3_)_2_·2H_2_O in DMSO-*d*_6_ (0.100 M) at r.t., many sets of ligand-based resonances were observed in the aromatic region of the ^1^H NMR spectrum (Fig. 2b). On annealing for two hours at 70 °C, this mixture coalesced into a single highly symmetric assembly (Fig. 2b and Supplementary Fig. 12). Annealing for eight hours at 100 °C afforded an identical spectrum, which implies that the assembly is stable under these conditions. The paddlewheel complex was characterized further by high-resolution electrospray-ionization time-of-flight mass spectrometry (HR-ESI-TOF-MS); a dominant species with a mass/charge ratio (*m/z*) that corresponds to the triply cationic paddlewheel mononitrate was observed (Supplementary Fig. 14). Finally, although the quality of our crystallographic data was low, X-ray crystallography confirmed the connectivity of the M_2_L_4_ paddlewheel complex (Fig. 2c).

Molecular dynamics simulations of the assembly of a simplified **L1** without the benzyl alcohol substituent (see the Supplementary Information for simulation details) revealed the formation of large clusters after 1 μs with an average number of ligands per cluster, *ȳ*, of 40 ± 20 (Fig. 2d). In agreement with simulation results from Yoneya and co-workers^50,51^, this result captures the early stages of the assembly process; the target M_12_L_24_ cages are not formed in high yield after 1 μs. Figure 2d (bottom) shows a representative M_12_L_24_ assembly obtained from the simulation. In the case of **L2**, the simulations yielded *ȳ* = 6.3 ± 0.5 after 1 μs with several of the target M_2_L_4_ paddlewheels present (Fig. 2e). Thus, our simulations suggest that the M_2_L_4_ paddlewheel forms more readily than the M_12_L_24_ cage within 1 μs. Collectively, these experimental data and precedents from Fujita and co-workers support the notion that ligands **L1** and **L2** form the target M_12_L_24_ and M_2_L_4_ assemblies, respectively, on thermal annealing.

### Formation of polyMOCs

Next we turned to the formation of polyMOCs **gel-1** and **gel-2** from polymeric ligands **PL1** and **PL2**, respectively, and Pd^2+^. Exposure of **PL1** to Pd(NO_3_)_2_·2H_2_O in DMSO-*d*_6_ at 23 °C resulted in the immediate formation of an opaque gel (Fig. 3a), which suggests the presence of large clusters^52^. The gel was annealed under conditions similar to those used to induce the self-assembly of free ligands; the annealing process was monitored by variable-temperature ^1^H MAS NMR (VT ^1^H MAS NMR) spectroscopy (Fig. 3a and Supplementary Fig. 24). Owing to the very broad ^1^H resonances in the MAS NMR spectrum of **gel-1** (Fig. 3a and Supplementary Fig. 24), we could not resolve the spectral changes on thermal annealing. However, the aromatic resonances observed for the annealed material (Fig. 3a, red spectrum) have the same chemical shifts as those observed in solution ^1^H NMR spectra of **L1** assemblies (Fig. 2a), and also soluble coordination polymers formed from mixing **PL1** with Pd^2+^ at a high dilution followed by annealing (Fig. 3a, black spectrum, and Supplementary Fig. 23). Although the majority of junctions in **gel-1** could be the target Pd_12_L_24_ cages (judging from the chemical-shift consistency with soluble analogues and the symmetric peak shape), we cannot confirm this conclusively from MAS NMR; cage fragments or larger clusters could yield similar spectra.

**Figure 1:**
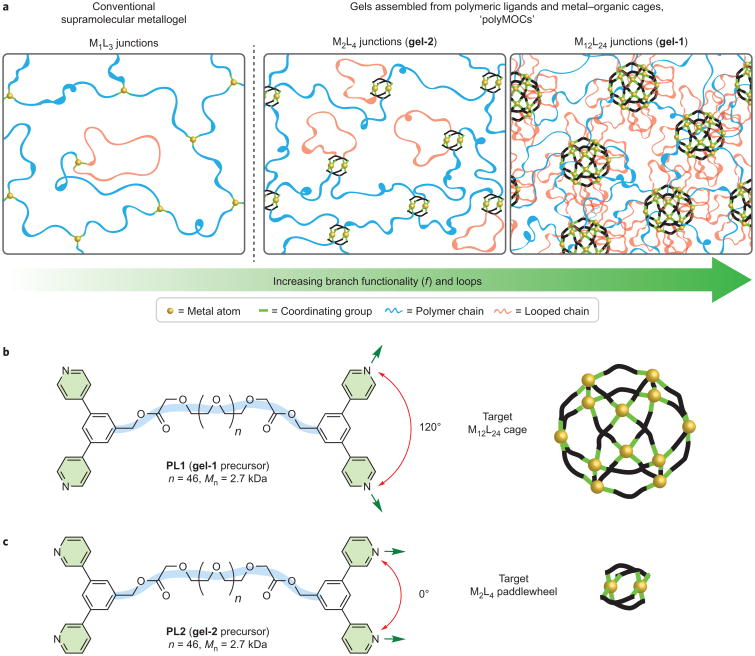
Design of polyMOCs with variable junction structures from isomeric polymer precursors **a** , Schematic representations of traditional supramolecular metallogels compared with the polyMOCs with M_2_L_4_ and M_12_L_24_ junctions proposed herein. The branch functionality, *f*, is the average number of chains (shown in blue) emanating from one junction that connect to another unique junction. Loop defects (shown in red) are polymer chains with both ends attached to the same metal atom or metal–ligand cluster. As the number of ligands per junction increases, both *f* and the fraction of looped chains are expected to increase. **b**, Chemical structure of bis-*para*-pyridine-terminated PEG **PL1** and a schematic of the M_12_L_24_ cage that is expected to arise from the assembly of 24 bis-*para*-pyridine ligands and 12 Pd^2+^ atoms. **c**, Chemical structure of bis-*meta*-pyridine-terminated PEG **PL2** and a schematic of the M_2_L_4_ paddlewheel that is expected to arise from the assembly of four bis-*meta*-pyridine ligands and two Pd^2+^ atoms. Exposure of **PL1** or **PL2** to Pd^2+^ yields isomeric polyMOCs **gel-1** or **gel-2**, respectively. *M*_n_, number-average molar mass.

SANS experiments were conducted to provide further support for the proposed structure of annealed **gel-1** (Fig. 3b). The SANS model that best fit the overall scattering curve (Fig. 3b, inset schematic) is a summed model of a power law at low scattering angles/longer distances, indicative of a long-range polymer network structure, and of the core-chain model at mid-to-high scattering angles, which describes the local gel nanostructure (that is, the polymer-bound junctions). Originally calculated by Hore *et al*.^53^ to describe a solid inorganic nanoparticle surrounded by the attached polymer chains in a nanocomposite system, the core–chain model fits well with the proposed **gel-1** structure of Pd_12_L_24_ cage junctions within a PEG network. The model fit to the SANS data provides a cage radius of 1.7 ± 0.2 nm with approximately 20 polymer chains emanating from and surrounding the cage core. These values agree well with the expected ∼1.8 nm cage radius reported by Fujita and co-workers^46^, and with the fact that we would expect 24 chains per cage if every cage formed perfectly. These SANS data provide strong evidence that the structure of **gel-1** is similar to that proposed above (Fig. 1a, far right).

Molecular dynamics simulations 1 μs after exposure of **PL1** to Pd^2+^ (see the Supplementary Information for the simulation details) revealed the presence of large clusters (*ȳ* = 21 ± 6) connected by highly extended polymer chains (Fig. 3c). This average cluster size (that is, the number of bis-pyridyl polymer end groups per cluster) agrees quite well with the experimental value observed by SANS, although we stress that, as for the assembly with the free ligands discussed above, after 1 μs the simulated **gel-1** does not reflect the reality of the thermally annealed network. Instead, we use simulations here to calculate *f* and the number of looped chains for non-annealed networks; these values will be important for comparison with mechanical property data (*vide infra*). The simulated cluster-size distribution in **gel-1** (Supplementary Fig. 26) was quite broad and contained some very large clusters with over 50 ligands (Fig. 3c). Given the relatively short polymer chains that link these clusters, a majority of the network chains (68%) are primary loops (Fig. 3c, red chains). These chains do not contribute to *f*, which leads to a calculated *f* of 6.7. Although this value is clearly well below the maximum possible value of 24, it is nonetheless higher than that possible for any traditional supra-molecular metallogel based on point-like metal junctions. As discussed below, this fact remains true although thermal annealing reduces *f* and induces even more loop-defect formation.

The properties of **gel-2** were strikingly different compared with those of **gel-1**. First, **gel-2** was translucent rather than opaque, which immediately suggested the presence of smaller junctions and a more homogeneous network (Fig. 3d). In **gel-2**, the MAS NMR spectra revealed a transformation similar to that observed for free ligand **L2**: on heating for one hour at 70 °C, the ligand-derived resonances coalesced and sharpened into single resonances that mapped closely onto the solution ^1^H NMR spectrum of the **L2**-based paddlewheels (Fig. 3d and Supplementary Figs 12 and 25). These data strongly suggest that the network junctions are converted into the target symmetric paddlewheels.

**Figure 2:**
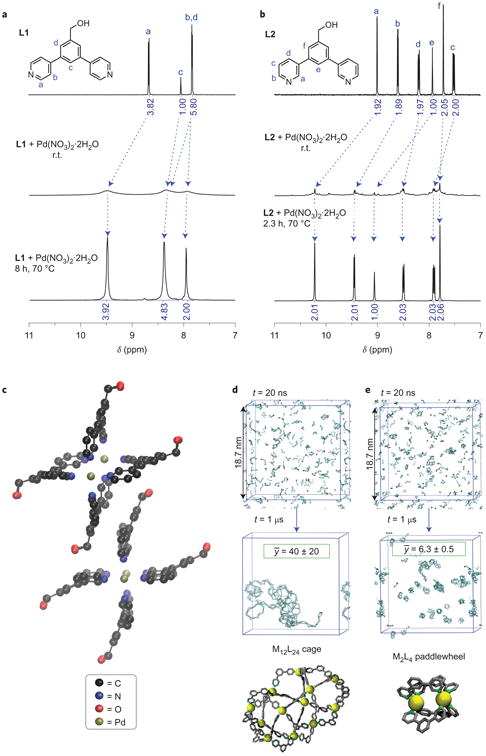
Solution self-assembly of junctions not bound to a polymer **a** , Aromatic regions of the solution ^1^H NMR spectra (400 MHz, DMSO-*d*_6_, 25 °C) of, from top to bottom, **L1**, the initial mixture of **L1** and Pd(NO_3_)_2_·2H_2_O prepared at r.t. and the same mixture after thermal annealing. **b**, Aromatic regions of the solution ^1^H NMR spectra (400 MHz, DMSO-*d*_6_, 25 °C) of, from top to bottom, **L2**, the initial mixture of **L2** and Pd(NO_3_)_2_·2H_2_O prepared at r.t. and the same mixture after thermal annealing. **c**, Single-crystal X-ray structure of ${\left(\text{L2}\right)}_{4}{\text{Pd}}_{2}^{∥}$. Crystals were obtained by vapour diffusion of ethyl acetate into DMSO-*d*_6_ at 23 °C. As a result of the significant disorder the quality of the structure was not suitable for the analysis of bond lengths and/or angles. We can confirm the paddlewheel connectivity of the complex as shown. **d**, Snapshots of the *in silico* assembly of an **L1** derivative without benzyl alcohol groups and Pd^2+^ after 1 μs at 77 °C initialized from a random configuration. *ȳ* = average number of ligands per cluster. Bottom: example of a simulated M_12_L_24_ cage. **e**, Snapshots of the *in silico* assembly of an **L2** derivative without benzyl alcohol groups and Pd^2+^ after 1 μs at 77 °C initialized from a random configuration. Bottom: example of a simulated M_2_L_4_ paddlewheel.

**Figure 3:**
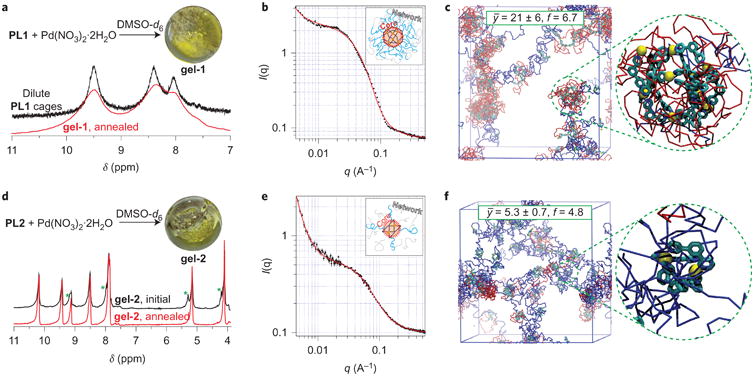
PolyMOC assembly and characterization **a** , Synthesis of **gel-1**. In black (dilute **PL1** cages): aromatic region of the solution ^1^H NMR spectrum of annealed cages with looped PEG chains derived from 1 and Pd^2+^ at a high dilution. In red: aromatic region of the ^1^H MAS NMR spectrum of annealed **gel-1**. **b**, SANS curve (black) for **gel-1** and schematic model used to fit (red) the SANS data. **c**, Snapshot of the *in silico* self-assembly of **gel-2** after 1 μs at 77 °C. Looped and non-looped polymer chains are shown in red and blue, respectively. The inset shows a representative loop-rich cluster. **d**, Synthesis of **gel-2**. ^1^H MAS NMR spectra of **gel-2** before (black) and after (red) annealing. Green asterisks highlight resonances that disappear or sharpen on annealing. **e**, SANS curve (black) for **gel-2** and schematic model used to fit (red) the SANS data. **f**, Snapshot of the *in silico* self-assembly of **gel-2** after 1 μs at 77 °C. Inset shows a representative M_2_L_4_ junction.

SANS data further support the structure of **gel-2**. As with **gel-1**, the best fit for the overall scattering curve is a summed model of a power law to describe the network structure and the core-chain model to describe the local nanostructure (Fig. 3e). From the fit, the calculated radius of the paddlewheel core in **gel-2** is 0.53 ± 0.05 nm, with approximately four polymer chains emanating from each paddle-wheel core. Again, these values agree quite well with what we would expect for a **gel-2** network architecture based on the crystal structure of the paddlewheel complex (Fig. 2c) and that four polymer chains should be connected to each junction.

As with **gel-1**, we used molecular dynamics simulations of **gel-2** to interrogate the network structure at the early stages of formation. In agreement with the data shown for free ligands, which suggest that M_2_L_4_ paddlewheels form more readily within 1 μs than M_12_L_24_ cages, simulations of the formation of **gel-2** after 1 μs revealed a preponderance of the target M_2_L_4_ paddlewheel assembly (Fig. 3f). The average cluster size in this case was *ȳ* = 5.3 ± 0.7 bis-pyridyl groups; the cluster distribution possessed a peak that corresponded to clusters containing four bis-pyridyl groups (Supplementary Fig. 26). As expected for the smaller junction size in **gel-2** compared with that in **gel-1**, only 25% of the polymer chains in **gel-2** were loops. The calculated *f* for **gel-2** was 4.8, which is greater than four because of the presence of some large clusters.

### Mechanical properties of polyMOCs

**Figure 4:**
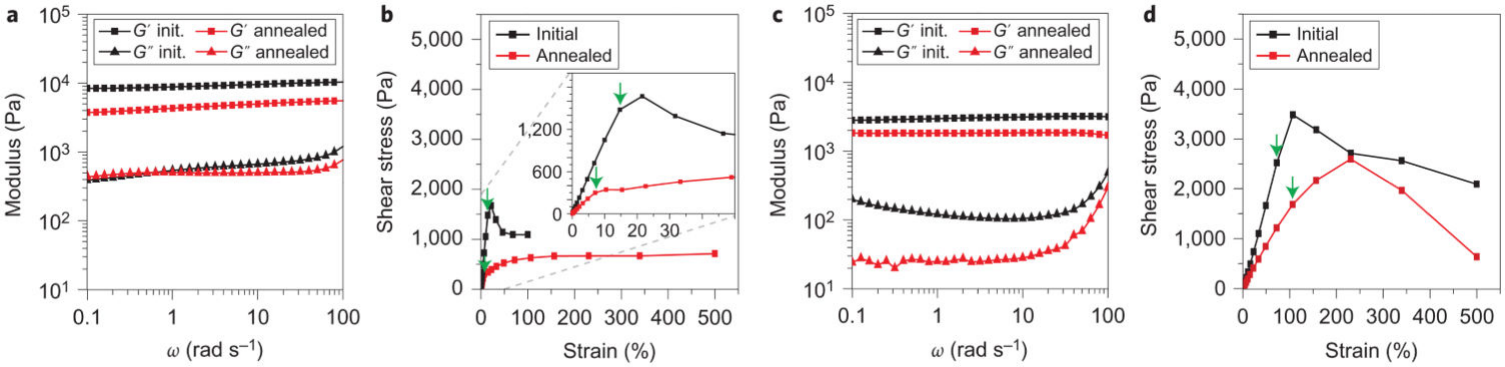
Room-temperature rheology of polyMOCs **a** , Frequency sweeps in oscillatory rheometry of **gel-1** samples at a 1.0% strain amplitude before (black) and after (red) thermal annealing for four hours at 80 °C. **b**, Stress versus strain plots before (black) and after (red) the thermal annealing of **gel-1**. **c**, Frequency sweeps in oscillatory rheometry of **gel-2** samples at a 1.0% strain amplitude before (black) and after (red) thermal annealing for four hours at 80 °C. **d**, Stress versus strain plots before (black) and after (red) the thermal annealing of **gel-2**.

Next, we used oscillatory rheometry to relate the mechanical properties of **gel-1** and **gel-2** to their network structures. First, the storage and loss moduli (*G′* and *G*″, respectively) of **gel-1** (5.9 wt% in DMSO-*d*_6_ (Fig. 4a)) were studied. Prior to thermal annealing, the high-frequency *G′* was 12 ± 3 kPa (Fig. 4a). Based on the phantom network theory of rubber elasticity, which relates *G′* to *f* and the mass density of elastically active polymer chains^32,54^, we estimate an *f* of 6.9 ± 1.6 (see the Supplementary Information for details of the calculation), which agrees well with the value obtained from simulations (Fig. 3c). Thermal annealing led to a 57% decrease in the high-frequency *G′* value to 5.2 ± 0.3 kPa, which corresponds to an *f* of 4.1 ± 0.1 (Fig. 4a, Supplementary Fig. 29 and Supplementary Table 1). To rationalize this decrease in *f* observed on annealing, we propose that annealing drives the fraction of very large clusters (that increase *f*) in the non-annealed **gel-1** towards the target cluster size of M_12_L_24_ and thus reduces *f*. Furthermore, because the target M_12_L_24_ cages cannot pack effectively around each other with relatively short PEG linkers (compared with the cage size) attached to every ligand, the vast majority of the polymer chains must either bridge the same two cages (double loops) or form primary loops. Although neither type of loop can be measured directly in these materials at this time^55,56^, the simulation data discussed above for pre-annealed materials suggest that the percentage of primary looped chains can, indeed, be very high. As we show below, the presence of such a large number of loop defects provides the opportunity to convert some of these defects into functional species through free-ligand replacement, which offers possibilities for functional network designs that cannot be realized in materials with a low *f* and fewer elastically inactive network defects.

After annealing, the yield stress of **gel-1** dropped by 87% from 2.1 ± 0.8 kPa to 0.26 ± 0.11 kPa and the yield strain decreased from ∼18% to ∼6.3% (Fig. 4b, Supplementary Fig. 30 and Supplementary Table 2). These results further suggest that **gel-1** consists of large clusters connected by highly extended PEG chains, the latter of which cannot bear large stresses. In future studies, increasing the PEG chain length could facilitate enhancements in the yield stress in **gel-1** analogues with potential decreases in *G′* offset by a decreased likelihood of primary-loop formation.

As expected, the mechanical properties of **gel-2** were quite different from those of **gel-1**. The measured *G′* for **gel-2** prior to annealing was significantly lower than that measured for **gel-1** (3.0 ± 0.5 kPa) (Fig. 4c, Supplementary Fig. 29 and Supplementary Table 1). On thermal annealing, a 37% decrease in the high-frequency *G′* value to 1.9 ± 0.2 kPa was observed, which, based on the phantom network theory, corresponds to an *f* of 2.13 ± 0.02 (Fig. 4c, Supplementary Fig. 29 and Supplementary Table 1). This value is close to the limiting value of *f* = 2 below which gelation cannot occur. As described for **gel-1**, we believe that annealing converts the large clusters in **gel-2** to the target M_2_L_4_. As fewer large clusters are formed initially in **gel-2** compared with **gel-1** (as observed in the simulations above), the corresponding decrease in *G′* on annealing is smaller.

The strain and swelling behaviours of **gel-1** and **gel-2** were clear indicators of the emergent bulk properties derived from junction self-assembly. Prior to annealing, the yield stress of **gel-2** (2.6 ± 0.4 kPa (Fig. 4d, Supplementary Fig. 30 and Supplementary Table 2)) was similar to that of **gel-1** (2.1 ± 0.8 kPa). However, although **gel-1** showed an 87% decrease in yield stress after annealing, the yield stress of **gel-2** decreased by only 31% to 1.8 ± 0.1 kPa. Furthermore, although the yield strain of **gel-1** decreased on annealing (Fig. 4d, Supplementary Fig. 30 and Supplementary Table 2), the yield strain of **gel-2** increased from ∼83 to ∼110% (Fig. 4d, Supplementary Fig. 30 and Supplementary Table 2). Gel-2 could withstand a more than 17-fold greater strain than could **gel-1**. Furthermore, **gel-2** absorbed 157 ± 9 times its own weight in DMSO after five days (Supplementary Fig. 31 and Supplementary Table 3). In contrast, the swelling ratio for **gel-1** was 23 ± 2. These data suggest that the average mesh size is much larger for **gel-2** compared with that for **gel-1**, and that the junctions within **gel-2** are potentially more dynamic. Indeed, when a sample of **gel-2** was cut into two pieces, it visibly healed on heating (Supplementary Fig. 32). Cuts in **gel-1** did not heal under the same conditions (Supplementary Fig. 32).

### Loop exchange in polyMOC **gel-1** with high *f*

**Figure 5:**
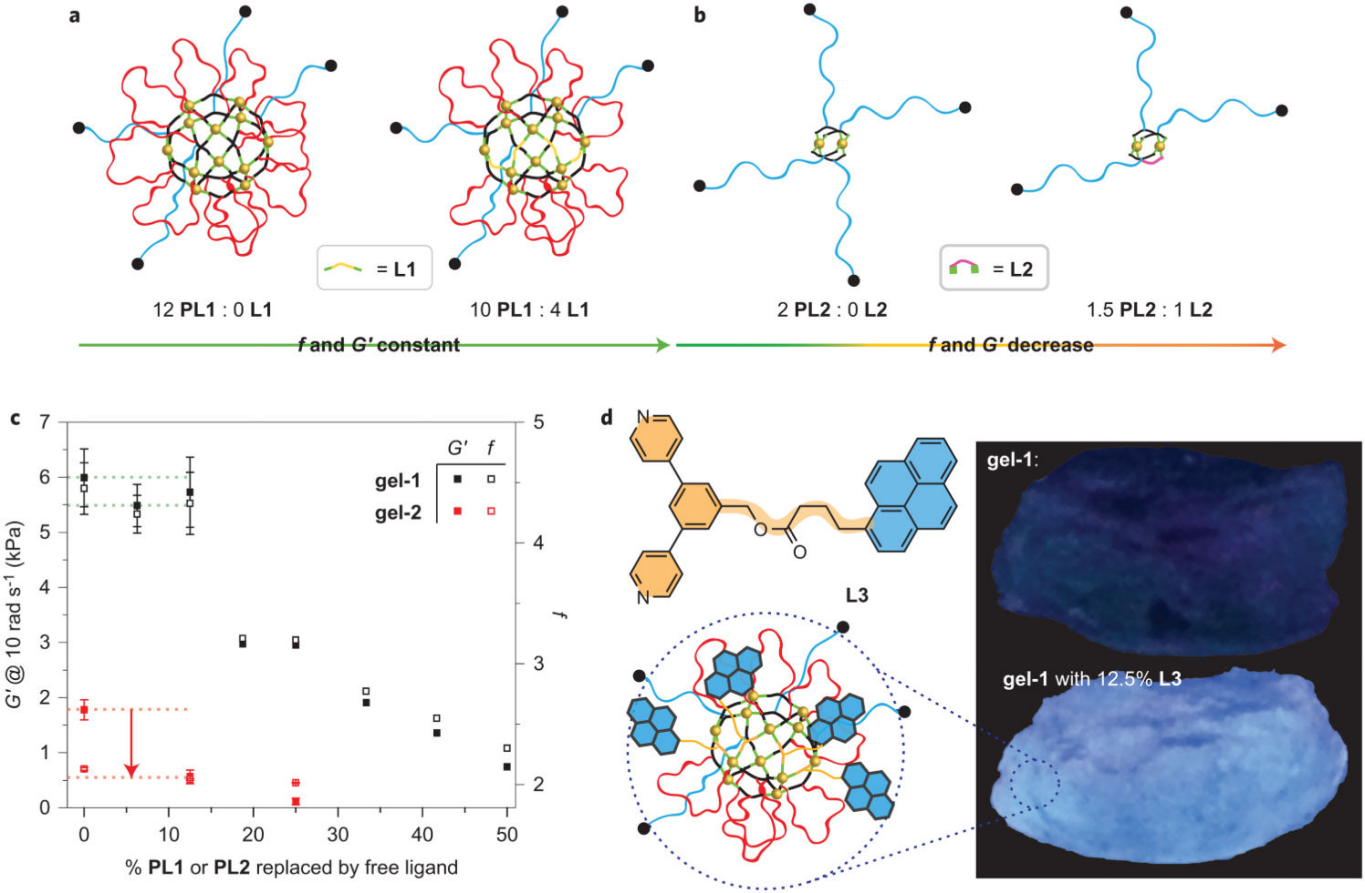
Loop-defect exchange in polyMOCs **a** , Schematic of representative junctions in polyMOC **gel-1** before (left) and after (right) substitution of **PL1** by 2 equiv. of **L1**. Primary loops are indicated in red, elastically active chains in blue and incorporated **L1** in orange. **b**, Schematic of representative junctions in polyMOC **gel-2** before (left) and after (right) substitution of **PL2** by 2 equiv. of **L2**. Primary loops are indicated in red, elastically active chains in blue and incorporated **L2** in purple. **c**, The effect of the percentage of polymer **PL1** or **PL2** replaced with the corresponding free ligands **L1** or **L2** on *G′* and the calculated *f* of polyMOCs **gel-1** and **gel-2**, respectively (Supplementary Table 4). **d**, The structure of pyrene-based ligand L3 (top left) and schematic of a representative junction in **gel-1** with 12.5% L3 added in place of **PL1** (bottom left). Photographs of **gel-1** (top right) and **gel-1** (bottom right) with 12.5% **PL1** replaced with L3 after extraction with excess DMSO (bottom). The polyMOCs were photographed under long-wavelength ultraviolet light.

The results above highlight how simple polymeric ligand design and the switch from *para* to *meta* bispyridine can translate into vastly different polyMOC properties; **gel-1** and **gel-2** behave as though they were different classes of materials (roughly covalent versus traditional supramolecular gels, respectively). Given the structure of **gel-1**, which features a large fraction of loops (68% from simulations of pre-annealed networks and 84% from *G′* measurements, an assumed maximal *f* of 24 and no other defects), compared with that of traditional gels, we wondered if it would be possible to replace selectively these loop defects with free ligands (dangling-end defects) that contain alternative functionality. Usually, the mechanical properties of gels (for example, *G′*) are extremely sensitive to loop and dangling-end defects that reduce *f*; the addition of even a small amount of free ligand would dramatically lower *G′*. Given the large loop fraction of **gel-1** and that *G′* is less sensitive to *f* for networks with increased *f*, we suspected that the incorporation of free ligands into **gel-1** could be possible with minimal or no net change in *G′* (Fig. 5a). In contrast, for **gel-2**, for which *f* is lower (∼2.13) and which has relatively fewer loops (∼46% based on *G′* and an assumption of no other defects), the introduction of free ligands should immediately reduce the network connectivity towards the limiting value of *f* = 2. In this case, *G′* should drop precipitously with the introduction of free ligand (Fig. 5b); such behaviour would also be expected in all other traditional gels with a low *f* and few network defects. This junction-engineering concept of the selective exchange of loop defects with functional dangling ends in a gel, with no net change in *G′*, would represent a feature of **gel-1** that, to our knowledge, has not been demonstrated in a polymer network.

To explore this possibility, we measured *G′* for analogues of **gel-1** and **gel-2** in which, during the gel preparation (see the Supplementary Information for the procedure), varying fractions of polymers **PL1** and **PL2** were replaced with 2 equiv. of free ligands **L1** and **L2**, respectively (Fig. 5c, filled squares). As before, these *G′* values were used to calculate the *f* values based on the phantom network theory (Fig. 5c, open squares (see the Supplementary Information for details)). When up to 12.5% of **PL1** was replaced with 2 equiv. of **L1**, *G′* and *f* were virtually unchanged. Although *G′* for **gel-1** begins to decrease rapidly as more free ligand is added, even with 50% of **PL1** replaced (a network concentration of 3.8 wt% or 45 mg ml^−1^ in DMSO-*d*_6_), the material retained a modulus comparable to that of traditional supramolecular metallogels with a substantially higher polymer content (network concentration of 10 wt% or 100 mg ml^−1^ in water^9^). The *f* value of **gel-1** with 50% free ligand was 2.29, which is similar to that of pristine **gel-2** with no free ligand. As predicted, the *G′* of **gel-2** dropped steeply (by 68 ± 9%) after only 12.5% of **PL2** was replaced, which confirms that network **gel-2** with a low *f* is more sensitive to network defects.

Having established that **gel-1** is much less sensitive to free-ligand defects than **gel-2**, we envisioned that additional functionality could be introduced to **gel-1** through defect engineering with a functional free ligand (L3 (Fig. 5d)). Although ligand replacement to introduce functionality has been explored thoroughly in the context of rigid three-dimensional networks (for example, MOFs^57^), the concept of free-ligand addition in place of loop defects in gels is a feature made possible by the structure of **gel-1**. Indeed, replacement of 12.5% of **PL1** with pyrene-based fluorescent ligand L3 during the gel preparation (see the Supplementary Information for details) provided a new polyMOC gel that exhibited blue fluorescence under long-wavelength ultraviolet light. This fluorescence persisted after continuous extraction of the gel with DMSO (∼66-fold excess) for two days (Fig. 5d and Supplementary Fig. 33); no detectable L3 was removed by extraction, which suggested that L3 was incorporated within the junctions of the polyMOC. The *G′* of L3-modified **gel-1** was within experimental error of the analogous **gel-1** with non-fluorescent ligand **L1** (Supplementary Fig. 34). This demonstration of ligand replacement in the junctions of **gel-1** opens exciting avenues for modular polyMOC synthesis; through the use of different free ligands, a range of mechanically uniform materials with distinct properties could be envisaged.

## Discussion

Herein we describe a novel class of polyMOC materials that feature self-assembled metal–ligand clusters as junctions connected by flexible polymer chains. A combination of MAS NMR, SANS, simulation and rheometry was used to study the structure and properties of these materials. These studies show that polyMOCs designed from compositionally identical but isomeric precursors can display a wide range of viscoelastic properties that spans from covalent-like gels to dynamic supramolecular gels. We demonstrate that in polyMOCs with large junctions and a high number of loop defects it is possible to replace selectively defects with functional free ligands to imbue the material with a novel function (in this case, fluorescence) without compromising mechanical integrity. Given the vast array of metal–ligand combinations that are known to provide discrete supramolecular assemblies, and the potential to incorporate many of these within the polyMOC paradigm, we anticipate the development of a range of new polyMOCs with robust, dynamic and otherwise unprecedented properties.

## Methods

### General synthesis of polyMOCs

To a 1 dram (3.7 ml) scintillation vial was added 20.25 mg (7.5 μmol) of macromer (1 or 2) and then 210.0 μl of DMSO-*d*_6_. In a 2 ml scintillation vial, a stock solution of Pd(NO_3_)_2_·2H_2_O in DMSO-*d*_6_ was prepared at a concentration of 22.2 mg Pd(NO_3_)_2_·2H_2_O per 1.00 ml DMSO-*d*_6_ (after vortexing for about one minute, a clear orange solution formed). This solution (90 μl) was transferred via micropipette to the solution of the macromer, and gelation was observed immediately, although the gel coloration was inhomogeneous. The headspace of the vial was purged briefly with argon, the vial was sealed and heated at 80 °C for four hours to give rise to a homogeneous light-yellow gel (translucent if derived from **PL2**, opaque if derived from **PL1**). The molarity of the macromer in the gel (in this case 24 mM) was determined by dividing the number of moles of the macromer used by the total volume of the gel, which accounts for the non-negligible contribution of the polymer to the total volume. All other methods and materials are described in the Supplementary Information.

### Characterization and other studies

The Supplementary Information also contains complete characterization of free ligands **L1**–L3, assemblies of **L1** and **L2** with Pd(NO_3_)_2_·H_2_O and **PL1** and **PL2** (Supplementary Figs 1 and 22), cryo-transition electron microscopy (cryo-TEM) images of linked cages derived from **PL1** and Pd(NO_3_)_2_·H_2_O at high dilutions (Supplementary Fig. 23), VT ^1^H MAS NMR of polyMOCs (Supplementary Figs 24 and 25), computational details (Supplementary Figs 27 and 28), details of SANS experiments and data fitting, complete rheometry data (Supplementary Figs 29 and 30, and Supplementary Tables 1 and 2), swelling data (Supplementary Fig. 31 and Supplementary Table 3), self-healing studies (Supplementary Fig. 32 and Supplementary Table 3), studies of the effect of the percentage of macromer replaced with free ligands **L1** or L3 on fluorescence, *G′* and *f* of the polyMOCs (Supplementary Figs 33 and 34, and Supplementary Table 4).

### Accession codes

The X-ray crystallographic data for the structure of the paddlewheel complex reported in this study are deposited at the Cambridge Crystallographic Data Centre (CCDC) under deposition number CCDC 1423278.

## Supplementary Material

supplemental-1

supplemental-2
