# Supplementary material for: Highly branched and loop-rich gels via formation of metal–organic cages linked by polymers
Source: Nat Chem. Author manuscript; Available in PMC 2017 May 5. (PMC5418868; doi:10.1038/nchem.2390)
Supplement: supplemental-1 [file NIHMS854916-supplement-supplemental-1.pdf]

nature chemistry
SUPPLEMENTARY INFORMATION
DOI: 10.1038/NCHEM.2390

Highly branched and loop-rich gels via formation of metal–organic cages linked by polymers

Aleksandr V. Zhukhovitskiy,[1] Mingjiang Zhong,[1] Eric G. Keeler,[1,2] Vladimir K. Michaelis,[1,2] Jessie E.P. Sun,[3] Michael J. A. Hore,[4] Darrin J. Pochan,[3] Robert G. Griffin,[1,2] Adam P. Willard,[1] Jeremiah A. Johnson[1]*

Affiliations
[1]Department of Chemistry, Massachusetts Institute of Technology, 77 Massachusetts Avenue, Cambridge, MA 02139, U.S.A.
[2]Francis Bitter Magnet Laboratory, Massachusetts Institute of Technology, 77 Massachusetts Avenue, Cambridge, MA 02139, U.S.A.
[3]Department of Materials Science & Engineering, University of Delaware, 201 DuPont Hall, Newark, DE 19716, U.S.A.
[4]Department of Macromolecular Science and Engineering, Case Western Reserve University, Cleveland, OH 44106, U.S.A.

correspondence to: jaj2109@mit.edu

This supporting information file includes:

Table of Contents
Materials and Methods
Supplementary Text
Supplementary References
Supplementary Figures S1 to S34 (including NMR, HRMS, and MALDI-TOF spectra, GPC chromatograms, fluorimetry, swelling and self-healing data, as well as computational and rheometry figures)
Supplementary Tables S1 to S4

© 2015 Macmillan Publishers Limited. All rights reserved

MATERIALS AND METHODS ... 3
MATERIALS ... 3
CHROMATOGRAPHY METHODS ... 3
SOLUTION NUCLEAR MAGNETIC RESONANCE SPECTROSCOPY METHODS ... 4
MAGIC ANGLE SPINNING NUCLEAR MAGNETIC RESONANCE SPECTROSCOPY METHODS ... 4
MASS SPECTROMETRY METHODS ... 5
MICROSCOPY METHODS ... 5
MOLECULAR DYNAMICS SIMULATION METHODS ... 5
CRYSTALLOGRAPHY METHODS ... 7
SMALL-ANGLE NEUTRON SCATTERING (SANS) METHODS ... 9
RHEOMETRY METHODS ... 10
FLUORIMETRY METHODS ... 11
GEL SWELLING METHODS ... 11
COMPUTATIONS OF NETWORK BRANCH FUNCTIONALITY F FROM G' VIA PHANTOM NETWORK THEORY OF RUBBER ELASTICITY[S10, S11] ... 11
SUPPLEMENTARY TEXT ... 13
MODIFIED SYNTHESES OF PREVIOUSLY REPORTED COMPOUNDS ... 13
SELF-ASSEMBLY OF THE Pd_{12}(**L1**)$_{24}$ CAGE AND Pd_2(**L2**)$_4$ PADDLEWHEEL ... 13
SYNTHESIS OF **L2**, **L3**, TELECHELIC PEG MACROMERS **PL1** AND **PL2**, AND FREE LIGAND-SUBSTITUTED POLYMOC GELS ... 14
SUPPLEMENTARY REFERENCES ... 16
SUPPLEMENTARY FIGURE S1 ... 17
SUPPLEMENTARY FIGURE S2 ... 18
SUPPLEMENTARY FIGURE S3 ... 19
SUPPLEMENTARY FIGURE S4 ... 20
SUPPLEMENTARY FIGURE S5 ... 21
SUPPLEMENTARY FIGURE S6 ... 22
SUPPLEMENTARY FIGURE S10 ... 26
SUPPLEMENTARY FIGURE S11 ... 27
SUPPLEMENTARY FIGURE S12 ... 28
SUPPLEMENTARY FIGURE S13 ... 29
SUPPLEMENTARY FIGURE S14 ... 30
SUPPLEMENTARY FIGURE S15 ... 31
SUPPLEMENTARY FIGURE S16 ... 32
SUPPLEMENTARY FIGURE S17 ... 33
SUPPLEMENTARY FIGURE S18 ... 34
SUPPLEMENTARY FIGURE S19 ... 35
SUPPLEMENTARY FIGURE S20 ... 36
SUPPLEMENTARY FIGURE S21 ... 37

© 2015 Macmillan Publishers Limited. All rights reserved

SUPPLEMENTARY FIGURE S22 38
SUPPLEMENTARY FIGURE S23 39
SUPPLEMENTARY FIGURE S24 40
SUPPLEMENTARY FIGURE S25 41
SUPPLEMENTARY FIGURE S26 42
SUPPLEMENTARY FIGURE S27 43
SUPPLEMENTARY FIGURE S28 44
SUPPLEMENTARY FIGURE S29 45
SUPPLEMENTARY FIGURE S30 46
SUPPLEMENTARY FIGURE S31 47
SUPPLEMENTARY FIGURE S32 48
SUPPLEMENTARY FIGURE S33 49
SUPPLEMENTARY FIGURE S34 50
SUPPLEMENTARY TABLE S1 51
SUPPLEMENTARY TABLE S2 52
SUPPLEMENTARY TABLE S3 53
SUPPLEMENTARY TABLE S4 54

Materials and Methods

Materials

All non-deuterated solvents, including anhydrous solvents in Sure/Seal™ containers, and polyethylene glycol (PEG, M_n = 2 kDa), and 4-(1-pyrenyl)butyric acid were purchased from Sigma-Aldrich®. Pyridine-3-boronic acid pinacol ester and pyridine-4-boronic acid pinacol ester were purchased from Ark Pharm, Inc. Palladium(II) nitrate dihydrate and tetrakis(triphenylphosphine)palladium were purchased from Strem Chemicals, Inc. All deuterated solvents were purchased from Cambridge Isotope Laboratories, Inc. All other reagents and solvents were purchased from VWR® or Sigma-Aldrich®. All purchased reagents and solvents were used as supplied unless otherwise noted. All air-sensitive reactions were executed using standard Schlenk techniques. All filtration *in vacuo,* unless stated otherwise, was carried out over MAGNA nylon filter disks (Maine Manufacturing, LLC) with 0.45 μm pore size. Spectra/Por® 7 standard regenerated cellulose dialysis tubing (8 kDa molecular weight cut-off (MWCO), 25.5 mm diameter) was purchased from Spectrum® Laboratories.

Chromatography methods

Liquid chromatography–mass spectrometry (LC/MS) were performed on an Agilent 1260 LC system equipped with an Advanced Materials Technology HALO® C18 high performance column. Solvent gradients consisted of mixtures of Milli-Q® water with 0.1% acetic acid

© 2015 Macmillan Publishers Limited. All rights reserved

(AcOH) and HPLC-grade acetonitrile. Mass spectra were obtained using an Agilent 6130 single quadrupole mass spectrometer.

Preparative high performance liquid chromatography (prep-HPLC) was performed on an Agilent Technologies 1260 Infinity system equipped with a ZORBAX 300SB-C18 PrepHT column (ID x Length = 21.2 x 150 mm; particle size = 5 µm). Eluent flow rate was 20 mL/min, and the eluent composition consisted of mixtures of nano-pure water with 0.1% acetic acid (AcOH) and HPLC-grade acetonitrile. The eluent gradient consisted of a linear ramp from 20% to 60% acetonitrile during 0—18 min, followed by a ramp to 100% acetonitrile during 18-20 min. Polymer samples were dissolved at a concentration of 100 mg/mL, and injected in 1.0 or 0.50 mL volumes. The instrument was controlled using the OpenLAB PrepLC software.

Column chromatography was performed on a Biotage® Isolera One with Accelerated Chromatographic Isolation™ flash chromatography system, using Biotage® KP-Sil SNAP cartridges at the recommended flow rates (*e.g.*, 50 mL/min for 100 g SNAP cartridge).

Gel permeation chromatography (GPC) measurements were performed in tetrahydrofuran (THF) using an Agilent 1260 Infinity system with variable-wavelength diode array (254, 450, and 530 nm) and a refractive index detector, guard column (Agilent PLgel; 5µm; 50 x 7.5 mm), and three analytical columns (Agilent PLgel; 5µm; 300 x 7.5 mm; 10^5, 10^4, and 10^3 Å pore sizes). The instrument was calibrated with low-dispersity polystyrene (PS) standards between 1.7 and 3150 kg/mol. All runs were performed at 1.0 mL/min flow rate at 25 °C. The number-average molar mass (M_n), weight-average molar mass (M_w), and dispersity index ($Đ = M_w/M_n$) of **PL1** and **PL2** were calculated by applying the conversion from polystyrene samples described by Sadao and Mori (values of t = 0.916, s = 1.21, derived for PEG, were used for **PL1** and **PL2**).[S1]

Solution nuclear magnetic resonance spectroscopy methods

^{1}H nuclear magnetic resonance (^{1}H NMR) and ^{13}C nuclear magnetic resonance (^{13}C NMR) spectra were recorded on two Bruker AVANCE-400 NMR spectrometers (NIH Grant # 1S10RR013886-01). Chemical shifts are expressed in parts per million (ppm), and splitting patterns are designated as s (singlet), d (doublet), t (triplet), m (multiplet), and b (broad). Scalar coupling constants *J* are reported in Hertz (Hz). MestReNova LITE v5.2.5-4119 software (Mestrelab Research S.L.) was used to analyze the NMR spectra. ^{1}H and ^{13}C NMR spectra were referenced to solvent peaks as reported in literature.[S2]

Magic angle spinning nuclear magnetic resonance spectroscopy methods

Variable temperature ^{1}H magic-angle spinning solid-state nuclear magnetic resonance (VT ^{1}H MAS NMR) spectra were recorded on a 11.7 T (500 MHz, ^{1}H) home-built NMR spectrometer (courtesy of Dr. David Ruben, Francis Bitter Magnet Laboratory-MIT). The gel samples immediately after mixing components were loaded via syringe into a 4 mm RevNMR-style zirconia rotor (60 µl fill-volume) which was sealed with a Kel-F cap to reduce the ^{1}H background signal. The spectra were collected using a spinning frequency ($\omega_{r/2}\pi$) of 10 kHz with 128 co-added transients and a recycle delay of 3 seconds. Sample temperatures were varied between 20 and 70 °C and spectra were collected every 5 minutes over a period of eight hours. ^{1}H spectra were referenced to solvent peaks as reported in literature.[S2]

© 2015 Macmillan Publishers Limited. All rights reserved

Mass spectrometry methods

Vide supra for LC/MS methods.

High-resolution mass spectrometry (HRMS) was obtained using either (1) a Bruker Daltonics® APEXIV 4.7 Tesla Fourier Transform Ion Cyclotron Resonance Mass Spectrometer (FT-ICR-MS) with a direct analysis in real time (DART) or electrospray ionization (ESI) ion sources (NSF Grant #CHE-0234877) or (2) the Bruker Impact HD q-TOF mass spectrometer coupled to a 1290 uHPLC (see ESI-TOF mass spectrum of Pd_2(**L2**)$_4$ paddlewheels below).

Matrix-assisted laser desorption ionization-time of flight mass spectrometry (MALDI-TOF) was carried out using a Bruker Daltonics® Omniflex® MALDI-TOF instrument, operated using the FlexControl™ version 1.1 software. The data was analyzed using XMass software. The instrument was operated in a reflectron mode (200 ns pulsed ion extraction; reflector voltage = 19.7 kV; lens voltage = 9 kV; detector gain = 20x) with positive ion detection (ion source voltage = 19.0 kV). The laser was operated at a 60-65% intensity at a sampling rate of 1 shot per 1.0 ns, and ~200-1000 shots were averaged to achieve the desired signal-to-noise ratio. The target had a "Scout 49" geometry. The samples were prepared as follows: first, the matrix solution was prepared by making a saturated solution of α-cyano-4-hydroxycinnamic acid (CHCA) in 1:1 water/acetonitrile with 1% trifluoroacetic acid (TFA). Polymer samples were dissolved at a concentration of 2.5-5 mg/mL in acetonitrile. To 40 μL of the matrix solution was added 4 μL the solution of the polymer sample. 0.5 μL of this solution was spotted in several locations on the MALDI target plate. The reconstituted solution of Calibration Mixture 2 from the Sequazyme™ Peptide Mass Standards Kit (Applied Biosystems, part # P2-314300) was diluted 1:24 (v/v %) with the matrix solution, and 0.5 μL of this solution was spotted over half of the sample spots for each sample on the MALDI target plate. These standards provided for internal mass spectrum calibration. In cases where ionization of the polymer sample was hindered by the presence of the standards, external standard calibration was employed.

Microscopy methods

Cryogenic transmission electron microscopy (cryo-TEM) was performed on holey carbon grids by plunge freezing in liquid ethane using a Gatan Cryo-Plunge3 instrument, a Gatan Liquid Nitrogen Single Tilt Holder, and a JEOL 2100 FEG microscope. The samples were prepared similarly to the standard preparation of polyMOCs (*vide infra*), except at a low concentration ([**PL1**] = 4.6 mM) to afford soluble hyperbranched network fragments. Thus, **PL1** (6.3 mg, 2.3 μmol) in a 2-mL scintillation vial was dissolved in 350 μL DMSO-d^6, and to it was added a solution of $Pd(NO_3)_2 \cdot 2H_2O$ (0.67 mg, 2.5 μmol) in 150 μL DMSO-d^6 via micropipette; the reaction mixture was heated at 70 °C for 1 d. 200 μL of the resulting solution was then dialyzed against Milli-Q® water (700 mL, 1 d) in a 8 kDa MWCO dialysis tubing (*vide supra*), affording 760 μL of aqueous solution, which was used directly (undiluted) for microscopy.

Molecular dynamics simulation methods

The simulated model system consisted of fully atomistic ligands and metal ions whose interactions were mediated by an implicit solvent. The effect of the implicit solvent is to (**1**)

© 2015 Macmillan Publishers Limited. All rights reserved

screen electrostatic interactions via a generalized reaction field method [***Tironi, I.; Sperb, R.; Smith, P.; van Gunsteren, W. A Generalized Reaction Field Method for Molecular Dynamics Simulations. J. Chem. Phys. 1995, 102, 5451***], and (**2**) to exert random impulsive forces on the atoms via Langevin dynamics [***Yun-yu, S.; Lu, W.; Van Gunsteren, W. On the Approximation of Solvent Effects on the Conformation and Dynamics of Cyclosporin A by Stochastic Dynamics Simulation Techniques. Mol. Simul. 1988, 1, 369–383***]. Following the simulation work presented by Yoneya and Fujita (Refs. 50 and 51 of the main text) we did not explicitly describe the presence of negative counter-ion species and utilized the cationic dummy atom (CaDA) model [***Pang, Y. Novel Zinc Protein Molecular Dynamics Simulations: Steps Toward Antiangiogenesis for Cancer Treatment. J. Mol. Model. 1999, 5, 196–202***] to describe Pd^{2+} and the metal-ligand coordination. In the CaDA model metal ligand binding is described empirically through Coulombic interactions between partial charges on the ligand molecules and those on a model Pd^{2+} complex consisting of a neutral Pd core bonded to four dummy atoms, each with a partial charge of +0.5 and arranged in a square planar geometry.

Simulations were carried out using GROMACS (version 5.02) [***Berendsen, H.J.C., van der Spoel, D. and van Drunen, R., GROMACS: A message-passing parallel molecular dynamics implementation, Comp. Phys. Comm. 91 (1995), 43-56***]. For the **L1**-type ligands the inter- and intra-molecular interactions, along with the Ligand-Pd^{2+} Van der Waals interactions were described using a standard model force field. The details of the force field, along the associated GROMACS topology files, were adapted directly from simulations of Yoneya and Fujita, which can be found in the Methods sections of Ref. 51 of the main text. Therefore, our model of the *para*-substituted bis-pyridine (**L1**) monomers differ from that described in reference 51 only in that our model includes both the repulsive *and* attractive contribution to the short-range Lennard-Jones potential (in reference 51 they use a modified GROMACS package that includes the repulsive-only WCA short range potential). The additional presence of Lennard-Jones attractions in our simulations results in a decrease in overall computational efficiency, but is otherwise expected to have a negligible effect on the results. To model the **L2**-type ligand (the *meta*-substituted bis-pyridine) we modify the **L1** model by exchanging the position of adjacent nitrogen and carbon atoms while retaining the force field parameters and partial charges of the original *para*- substituted ligand (see Supplementary Figure S27). Reorganizing atoms in this manner allows us to isolate the influence of the Pd-N bite angle on the the metal-ligand complexes.

In the gel-forming system each ligand is bound to a partner ligand via long and flexible polymer chains. An explicit model of such a system dramatically increases the number of atoms being simulated and also introduces a long timescale relaxation process associated with chain entanglement. Here we have utilized an efficient compromise in which the effect of the flexible polymer linker is described implicitly, in the form of a ligand-ligand pair potential. The pair potential is meant to mimic the effect of the polymer linker on the relative positions of connected pairs of ligands. The effect is primarily entropic and is, as we have treated it, directly related to the statistics that govern the end-to-end distance of the isolated polymer.

We modeled the effect of flexible polymer linker through the addition of a pair potential, $w(r)$, acting between the bridging carbon atoms of pairs of ligand molecules (those with partial charge of -0.162 in Fig. S27). In particular we took $w(r)$ to be equal to the potential of mean force governing the end-to-end distance of a model polymer linker. This pair potential was generated based on simulations of an idealized version of the PEG linker. Given that the persistence length of PEG, $l = 3.8$Å,[S3] is approximately equal to the monomer size we modeled

© 2015 Macmillan Publishers Limited. All rights reserved

configurations of the 2.2kDa linker as a three-dimensional self-avoiding random walk with steps of length l randomly distributed around a unit sphere. By sampling the statistics of this idealized model polymer we determined the end-to-end PMF and tabulated it for use as a user-input pair potential in GROMACS. Supplementary Figure S28 contains a plot of the potential of mean force used to model the 2.2kDa linker.

The potential used to describe the flexible ligand-ligand polymer linker imparts forces that are much smaller than those involved in ligand-metal coordination. The distribution of cluster sizes is therefore quite insensitive to the details of $w(r)$. However, because this potential imposes spatial correlations on specific pairs of ligands, the network-forming properties system can be sensitive to the specific details of $w(r)$. The sensitivity is most pronounced in the statistics of inter-MOC connectivity where the probability for loop formation can be controlled by varying the shape of $w(r)$. For instance, $w(r)$ can be chosen to mimic a very short linker (keeping ligand pairs very close) so that loop formation is enhanced or to mimic a long linker (leveraging the entropic driving force that prevents configurations with very small end-to-end distances) so that loop formation is reduced. Our procedure is approximate but gives rise to ligand-ligand correlations that are in reasonable agreement with our physical expectations.

Simulations consisted of 96 metal ions and 192 ligands (or 96 macromers), enough to form four fully assembled $M_{12}L_{24}$ type clusters, in a cubic, periodically replicated simulation cell with side length 18.7nm. Initial configurations were generated by randomly distributing ligands and metal ions subject to the constraint that the initial separation between any two species be greater than 1.5nm. For the polyMOC formation, **PL1** and **PL2** were placed randomly but at an initial fixed distance of 2.6 nm for the 2.2kDa PEG chain. Simulations were carried out in the NVT ensemble with a 2 fs time step. For each ligand model (4 in total, i.e., **L-*para***, **L-*meta***, and **PL1** and **PL2**) we generated 20 individual trajectories. Each trajectory consisted of an initial 1 ns equilibration run at a temperature of 500K, followed by a 1 μs production run at a temperature of 350 K. The details of the implicit solvent and the thermostat were identical to those described in Ref. 52.

The results presented in Supplementary Figure S26A of the main text indicate that the **L-*para*** ligands tend to form large and sometimes system-spanning clusters. To explore the effect of concentration on the formation of very large ligand-metal clusters we carried out a set of simulations in which 192 **L-*para*** ligands and 96 metal ions were placed in a larger periodically replicated cubic cell, one with side length 30 nm. In Supplementary Figure S26B we present the distribution of cluster sizes, $P(y)$, that emerged from this 'low concentration' simulation compared to that of the high concentration simulation (the latter is an excerpt of the data plotted in Supplementary Figure S26A). To facilitate a side-by-side comparison, each curve has been normalized over the same increment, specifically $0 \leq y \leq 50$. At low concentration we observe a significantly reduced probability for observing clusters with $y > 50$, and in fact the distribution of cluster sizes for $y \leq 50$ are quite similar for the low and high concentration simulations. This indicates that the presence of large clusters is either the result of exceeding a percolation threshold in ligand concentration or a preferential stabilization of such clusters due to self-interaction through the periodic boundaries of the system.

Crystallography methods

Low-temperature (100 K) diffraction data (φ- and ω-scans) were collected on a Bruker X8 Kappa Duo four-circle diffractometer coupled to a Smart Apex2 CCD detector, with Mo K_α

© 2015 Macmillan Publishers Limited. All rights reserved

radiation (λ = 0.71073 Å) from an *IμS* micro-source. The diffractometer was purchased with the help of funding from the National Science Foundation (NSF) under Grant Number CHE-0946721. The structure was solved by direct methods using SHELXS[S4] and refined against F^2 on all data by full-matrix least squares with SHELXL-97[S5] following established refinement strategies[S6].

The final cif file was checked using the IUCr checkCIF routine, and below, we list the Alerts of level A and B as they appear in the output checkCIF file and the justification for each.

Alert level A

SHFSU01_ALERT_2_A The absolute value of parameter shift to su ratio > 0.20 Absolute value of the parameter shift to su ratio given 6.596 Additional refinement cycles may be required.
Author Response: Structure refinement is not complete. Data are of low quality and so is the structure. Paddlewheel connectivity is confirmed but not much else can be concluded from this structure.

PLAT080_ALERT_2_A Maximum Shift/Error .... 6.60 Why ?
Author Response: Structure refinement is not complete. Data are of low quality and so is the structure. Paddlewheel connectivity is confirmed but not much else can be concluded from this structure.

Alert level B

PLAT201_ALERT_2_B Isotropic non-H Atoms in Main Residue(s) .... 1 Report
Author Response: Structure refinement is not complete. Data are of low quality and so is the structure. Paddlewheel connectivity is confirmed but not much else can be concluded from this structure.

PLAT430_ALERT_2_B Short Inter D...A Contact O10N .. O2A .. 2.74 Ang.
Author Response: Structure refinement is not complete. Data are of low quality and so is the structure. Paddlewheel connectivity is confirmed but not much else can be concluded from this structure.

PLAT601_ALERT_2_B Structure Contains Solvent Accessible VOIDS of . 192 Ang3
Author Response: Structure refinement is not complete. Data are of low quality and so is the structure. Paddlewheel connectivity is confirmed but not much else can be concluded from this structure.

PLAT934_ALERT_3_B Number of (Iobs-Icalc)/SigmaW > 10 Outliers .... 3 check
Author Response: Structure refinement is not complete. Data are of low quality and so is the structure. Paddlewheel connectivity is confirmed but not much else can be concluded from this structure.

PLAT975_ALERT_2_B Check Calcd Residual Density 0.62A From O4S 1.54 eA-3
Author Response: Structure refinement is not complete. Data are of low quality and so is the structure. Paddlewheel connectivity is confirmed but not much else can be concluded

© 2015 Macmillan Publishers Limited. All rights reserved

from this structure.

Small-angle neutron scattering (SANS) methods

Small-angle neutron scattering (SANS) measurements were performed at the National Institute of Standards and Technology (NIST) Center for Neutron Research (NCNR) (Gaithersburg, MD, USA). The scattered neutron intensity was measured as a function of scattering variable q, where $q = (4\pi/\lambda)\sin(\theta/2)$ and θ is the scattering angle. The beam was monochromated to a wavelength, λ, of 6 Å. Three sample-to-detector distances of 1 m, 4 m, and 13 m were used to cover a total q range of 0.004 to 0.5 $Å^{-1}$. 400 µL of each sample (gels prepared at 3.54 wt. % of polymer network) was loaded into titanium sample cells with a 1 mm path length. Experiments were performed on the NGB 30 m SANS instrument. Collected data were reduced and analyzed using the SANS macros package provided by the NCNR[S7]. The resulting data were placed on an absolute scale and corrected for background electronic noise, detector inhomogeneity, and empty cell scattering using standard techniques.

Scattering data for both samples were fit using a sum of two models, the power law model and the core chain model. The power law model is primarily used to show the presence of a larger entangled network, and describes the scattering intensity as $I(q) = Aq^{-n}$. The core-chain model, due to Hore *et al.*[S8], is a slightly modified version of the reported model.[S9] The original Hore core-shell-chain model in ref. 2 was used to describe an inorganic iron oxide core with a shell layer of dense polymer brush, surrounded by grafted polymer chains with excluded volume. Here, the shell layer element is omitted but the model remains intact otherwise. In addition, the core in the present system is not inorganic entirely, but a mixed composition of Pd and bis-pyridine ligand. Most importantly, the model does not assume that the chains are Gaussian, and allows the excluded volume of the chains to vary. For this reason, Debye functions are omitted in favor of a more detailed description of polymer chain scattering.

The scattering intensity is calculated from the sum of the spherical core form factor, core-chain form factor correlations, chain-chain correlations, and the form factor of a polymer chain with excluded volume. The form factor amplitude of the spherical core is given by $F_A(q)$,

$$F_A(q) = \left[(\rho_{core} - \rho_{solvent})V_{core}\frac{3j_1(qr_{core})}{qr_{core}}\right] \quad (0.1)$$

where j_1 is a spherical Bessel function of order 1, r_{core} is the radius of the paddlewheel or cage, V_{core} is the volume of the paddlewheel or cage, ρ_{core} is the scattering length density (SLD) of the paddlewheel or cage, and $\rho_{solvent}$ is the SLD of the solvent.

Scattering from polymer chains is described by the form factor amplitude and form factor of the polymer chains, $F_B(q)$ and $P_B(q)$, respectively. Note that because polymer chains are fractal in nature, the form factor is a separate function from the form factor amplitude. The functions are given by,

$$F_B(q) = \frac{1}{2\nu U^{1/2\nu}}\gamma\left(\frac{1}{2\nu},U\right) \quad (0.2)$$

$$P_B(q) = \frac{1}{\nu U^{1/2\nu}}\gamma\left(\frac{1}{2\nu},U\right) - \frac{1}{\nu U^{1/\nu}}\gamma\left(\frac{1}{\nu},U\right) \quad (0.3)$$

where the lower incomplete gamma function reads

© 2015 Macmillan Publishers Limited. All rights reserved

$$\gamma(d,U)=\int_0^U dt(e^{-t}t^{d-1}) \tag{0.4}$$

The parameter $U = q^2 a^2 N^{2\nu}/6$ contains the scattering variable q, the statistical segment length of the polymer chain (a), the degree of polymerization of the chain (N), and the excluded volume parameter ν.
The total macroscopic scattering cross section for N_p/V density of nanoparticles with N_g grafted polymer chains per particle, including the power law term, is then expressed as

$$\frac{d\Sigma(q)}{d\Omega} = Aq^{-n} + \frac{N_p}{V}\begin{bmatrix} F_A(q)^2 + N_g V_B(\rho_{chain} - \rho_{solvent})F_A(q)F_B(q) \\ +N_g(N_g - 1)V_B^2(\rho_{chain} - \rho_{solvent})^2 F_B(q)E_A(q)^2 F_B(q) + N_g V_B^2(\rho_{chain} - \rho_{solvent})^2 P_B(q) \end{bmatrix} S_I(q) + B \tag{0.5}$$

where $E_A = j_0(qr_{core})$ is a spherical Bessel function, and B is the constant incoherent background. The radius of gyration for the chains surrounding the paddlewheel or cage is calculated from the parameters of Eq. (1.5) as

$$R_g^2 = \frac{N^{2\nu}a^2}{(2\nu+1)(2\nu+2)} \tag{0.5}$$

The SLDs for the core of each sample were calculated using an average of the PEG and palladium SLD on the basis of the composition of the two components. Using the NCNR SLD calculator, the bis-pyridine SLD was 1.98 x 10^{-6} / $Å^2$, the Pd SLD was 4.02 x 10^{-6} / $Å^2$, and DMSO-d6 had an SLD of 5.28 x 10^{-6} / $Å^2$. The composition of the components, calculated on the basis of the density and mass for paddlewheel and cage gels (**gel-2** and **gel-1**, respectively), was 70% ligand and 30% Pd yielding an initial SLD of 2.59 x 10^{-6} / $Å^2$. The initial SLD does not take into account the possible presence of PEG or DMSO within the core, and so is only an initial approximation. The calculated radii, resulting from the core-chain model fits, for the paddlewheel and cage structures were 0.55 ± 0.054 nm and 1.70 ± 0.25nm, respectively. The calculated number of ligands, also from the core-chain model fits, for the paddlewheel and cage structures, N_g, were approximately 4 and 20, respectively. The excluded volume parameter (ν), calculated from the core-chain model fit, for the paddlewheel gel and cage gel, were 0.574 and 0.595, respectively. A value of ν that is close to 0.6 is indicative of a swollen polymer chain (*i.e.*, $R_g \sim N^{0.6}$). The radius of gyration (R_g) calculated using Eq (1.6) with paramaters obtained from the core-chain model fits were 0.49 nm and 0.45 nm, respectively, for paddlewheel and cage gel.

We acknowledge the support of the National Institute of Standards and Technology, U.S. Department of Commerce, in providing the neutron research facilities used in this work. This work utilized facilities supported in part by the National Science Foundation under Agreement No. DMR-0944772. This manuscript was prepared under cooperative agreement 70NANB12H239 from NIST, U.S. Department of Commerce. The statements, findings, conclusions, and recommendations are those of the authors and do not necessarily reflect the view of NIST or the U.S. Department of Commerce.

Rheometry methods

© 2015 Macmillan Publishers Limited. All rights reserved

Frequency sweep and strain sweep experiments were performed on an Anton Paar MCR 301 rheometer. The rheometer was outfitted with a Peltier heating system with an environmental enclosure for temperature control. A disposable parallel-plate geometry (radius = 12 mm) was used and coupled with a disposable bottom plate, with the typical gap of 1.00 mm between the two plates. Frequency sweep experiments were performed from 0.1 to 100 rad/s at 1% strain, which was first confirmed to be in the linear viscoelastic regime using strain sweep experiments. Gel samples were prepared either on the plate *in-situ* or in 1-dram vials (*vide infra*) and subsequently transferred onto the rheometer. Experiments were performed at 25 $^{\circ}$C and the evaporation of solvent (DMSO-d^6) was negligible within the typical measurement time (< 1 hour).

Fluorimetry methods

Fluorimetry was carried out using Fluorolog®-3 spectrofluorometer from Jobin Yvon Horiba using the DataMax for Windows™ driving software. The following parameters were used during the fluorimetry: (1) integration time = 0.25 s; (2) increment = 1 nm; (3) excitation wavelength: 340 nm; (4) Detector HV S = 950 V and R = 0 V; bandpass slits: excitation1 = 3.000 nm; emission1 = 5.000 nm. The data was analyzed using OMNIC™ software and plotted in OriginPro 8.5. The samples for fluorimetry were prepared by depositing a fragment of the gel into a cylindrical hole (2 mm diameter x 0.9 mm depth) within a sample holder, sandwiching the sample holder with the gel between two square glass cover slips to stabilize the gel and prevent solvent evaporation, and using the front-face geometry to collect the fluorescence.

Gel swelling methods

Two sets of gels (3 gels per set) were prepared in tared 1-dram vials – one set from macromer **PL1** and the other set from macromer **PL2** – following the general polyMOC synthesis method (*vide infra*), except at 0.500 x the scale: i.e., 10.13 mg of macromer was used to prepare gels with [macromer] = 24 mM (Supplementary Figure S31). To each gel was added 3.8 mL DMSO, and the gels were allowed to stand at RT for 5 d, at which time the excess DMSO was removed via syringe, and any residual DMSO was wicked away by gently dabbing the gels in the vials with Kimwipes®. Vial inversion tests confirmed the materials remained in the gel state (see Supplementary Figure S31). The gels in the vials were weighed, and the swelling ratio for each was determined by diving the mass of the swollen gel by the dry mass of the network (dry mass of the network = mass of macromer + mass of $Pd(NO_3)_2 \cdot 2H_2O$ = 10.13 mg + 1.00 mg = 11.13 mg) (Supplementary Table S3). Averages and standard deviations were computed for the three trials.

Computations of network branch functionality f from G' via phantom network theory of rubber elasticity[S10, S11]

A measurement of $G' \sim |\boldsymbol{G}|$ (*i.e.*, $G' >> G''$) allows us in principle to compute f in our polyMOCs and thereby validate the conclusions derived from the simulations. The phantom network theory was deemed most appropriate for the analysis of our polyMOCs, because it explicitly relates $|\boldsymbol{G}|$ and f:[S11]

© 2015 Macmillan Publishers Limited. All rights reserved

$$|\boldsymbol{G}| = \frac{\rho RT}{M_{chain}}\left(\frac{f-2}{2}\right),$$

where ρ is the mass density of the elastically active polymer chains, R is the universal gas constant (8.31446•10^6 cm^3•Pa•K^{-1}•mol^{-1}), T is the temperature (in this case 298.15 K), M_{chain} is the number-average molecular weight (M_n) of the polymer chain separating the junctions (taken here to be equal to M_n of the macromers = 2700 g/mol).[S11] Crucially, ρ is not known *a priori* because an unknown fraction of the polymer chains may form primary loop defects (and therefore not contribute to ρ (dangling chain ends were not observed by NMR and can therefore be ignored). A key realization is that to a first-order approximation (i.e., accounting only for primary loops) ρ scales linearly with the true f of the network – in other words a deviation of f from f_{ideal} by x % is expected to yield the same x % deviation of ρ from ρ_{ideal}. Thus, we have

$$\rho = \frac{f}{f_{ideal}}\rho_{ideal},$$

and substitution of the expression for ρ into the expression for $|\boldsymbol{G}|$ yields

$$|\boldsymbol{G}| = \frac{\rho_{ideal}\cdot RT}{f_{ideal}\cdot M_{chain}}(f-2).$$

In the case of polyMOCs derived from **PL1** or **PL2**, where no macromer is replaced with free ligand **L1** or **L2**, f_{ideal} is taken to be 24 or 4, respectively, values expected for $M_{12}L_{24}$ or M_2L_4 cages, and consistent with $\bar{y}$ at 1 µs computed above for the corresponding networks (21 ± 6 and 5.3 ± 0.7, *vide supra*). When a fraction r of macromer **PL1** or **PL2** is replaced with **L1** or **L2**, respectively, new f_{ideal} becomes equal to (1 – r) • (f_{ideal} when r = 0) because the free ligands do not contribute to the branch functionality: i.e., f_{ideal} = (1 – r) • 24 for **gel-1**, and f_{ideal} = (1 – r) • 4 for **gel-2**.

G' and *G"* at ω = 10 rad/s were measured for all polyMOCs with varying fractions r of macromer replaced with **L1** or **L2** (Supplementary Table S4). Thus, when *G'* >> *G"*, as is the case for all of our polyMOCs at ω = 10 rad/s, *G'* can be used in place of |***G***|. Note: for the comparison of f for **gel-1** and **gel-2** before and after annealing, *G'* at ω = 100 rad/s was utilized. A sample calculation is provided below for a **gel-1** gel where r = 0.125. The volume of the gel is the added volume of DMSO-d^6 and the polymer network (the latter was estimated to be the same as that of PEG with M_n = 2000, which was measured to be 0.8273 mL/g).

$$G'@10\frac{rad}{s} = 4762.1\ \text{Pa} = \frac{\left(\frac{\text{mass of polymer}}{\text{volume of gel}}\right)\cdot 8.31446 \bullet 10^6\ \text{mL} \bullet \text{Pa} \bullet \text{K}^{-1} \bullet \text{mol}^{-1} \bullet 298.15\ K}{(1-0.125)\bullet 24\cdot 2700\frac{\text{g}}{\text{mol}}} \bullet (f-2) = \left(\frac{0.01772\ \text{g}}{0.01772\ \text{g} \bullet 0.8273\ \text{mL/g} + 0.300\ \text{mL}}\right) \bullet 43720.6\ \text{mL} \bullet \text{Pa} \bullet \text{g}^{-1} \bullet (f-2) = 2462.1\ \text{Pa} \bullet (f-2)$$

$$\therefore f = 2 + \frac{4762.1\ \text{Pa}}{2462.1\ \text{Pa}} = 3.93$$

© 2015 Macmillan Publishers Limited. All rights reserved

Supplementary Text

Modified Syntheses of Previously Reported Compounds

***(3,5-di(pyridin-4-yl)phenyl)methanol* L1**.[S12] To a 20-mL microwave vial equipped with a magnetic stir-bar were added 3,5-dibromobenzyl alcohol (0.442 g, 1.66 mmol), pyridine-4-boronic acid pinacol ester (0.750 g, 3.66 mmol), potassium carbonate (2.297 g, 16.6 mmol), and tetrakis(triphenylphosphine)palladium (0.192 g, 0.166), and the vessel was crimped shut, evacuated and refilled with nitrogen three times. To the vessel via syringe were added anhydrous *N,N*-dimethylformamide (DMF, 13.5 mL) and then de-ionized water (0.280 mL, 15.5 mmol) which had first been sparged with argon for 30 min. The vessel was briefly evacuated until bubbling was observed, and then refilled with nitrogen, and briefly sparged with argon (10-15 sec). The vessel was heated in the oil bath for 60 h at 100 °C, with precipitated palladium black observed after 24 h. At the conclusion of the reaction, the vessel was brought to RT, and the contents of the vessel were filtered *in vacuo* over tightly-packed Celite® 545 on a medium-porosity glass frit, rinsing the flask and Celite with chloroform (3 x 50 mL). The filtrate was subjected to washes with water (5 x 150 mL), followed by drying over Na_2SO_4, filtration over a medium-porosity frit, and concentration *via* rotary evaporation. The crude material was subjected to chromatography on silica gel (CH_2Cl_2/MeOH step gradient: 9.8:2 → 9.5:0.5 → 9:1; **L1** eluted during the second step, R_f = 0.087 in CH_2Cl_2/MeOH = 9.5:0.5). The fractions containing the desired product were combined, concentrated by rotary evaporation, and triturated with CH_2Cl_2 (3 x 1 mL) quickly to remove traces of a co-eluting, but more readily soluble in CH_2Cl_2 impurity. The CH_2Cl_2 rinsings were concentrated by rotary evaporation and triturated with tetrahydrofuran (THF, 4 x 1 mL) quickly. The two portions of triturated solids were combined and dried *in vacuo* affording **L1** (0.2035 g, 46.7 % yield, ≥99% pure by LCMS) as a white to faint-yellow powdery solid. ^{1}H NMR (400 MHz, DMSO-d^6, 25 °C): δ 8.68 (dd, J = 4.5, 1.6 Hz, 4H), 8.06 (t, J = 1.7 Hz, 1H), 7.87-7.80 (m, 6H), 5.40 (t, J = 5.7 Hz, 1H), 4.68 (d, J = 5.4 Hz, 2H). ^{13}C NMR (100 MHz, DMSO-d^6, 25 °C): δ 150.26, 146.75, 144.72, 138.06, 125.58, 123.77, 121.49, 62.57. FT-ICR-DART HRMS: calcd. for $C_{17}H_{14}N_2O$ $[M+H]^+$, most abundant *m/z* = 263.1179; found, 263.1160.

Polyethylene glycol (PEG)-diacid (M_n = 2.2 kDa) was synthesized following the general protocol disclosed in patent US 8,067,505 B2[S13] for the synthesis of PEG diacid from PEG diol, except beginning with PEG-diol (M_n ~ 2.0 kDa) and using sodium tert-butoxide instead of potassium tert-butoxide (the former formed a dispersion in 1:1 tert-butanol/toluene and was transferred via syringe with a 16-gauge needle). Product was isolated as a powdery white solid in 89% (7 g scale) or 93% (3.5 g scale) yields. ^{1}H NMR (400 MHz, DMSO-d^6, 25 °C): δ 13.35–12.01 (b, 2H), 4.01 (s, 4H), 3.71–3.43 (m, 184H). MALDI-TOF: M_n = 2.27 kDa; calcd. for $C_{98}H_{194}O_{52}$ $[M+Na]^+$, most abundant *m/z* = 2227.2; found, 2227.2. M_n (^{1}H NMR) = 2.16 kDa. M_n = 2.2 kDa was employed in the calculations for reactions involving this **PEG-diacid**.

Self-assembly of the Pd_{12}(**L1**)$_{24}$ cage and Pd_2(**L2**)$_4$ paddlewheel

Pd_{12}(L1)$_{24}$ cage. To a 2-mL vial with **L1** (13.13 mg, 0.05006 mmol) dissolved in 366.7 µL of DMSO-d^6 was added via micropipette a solution of $Pd(NO_3)_2 \cdot 2H_2O$ (6.67 mg, 0.02503 mmol) in

© 2015 Macmillan Publishers Limited. All rights reserved

133.3 μL of DMSO-d^6. The head-space of the vial was briefly purged with argon, the vial was closed with a screw-cap, and the resultant mixture was immediately vortexed, giving rise to a light-yellow liquid with small gelatinous pieces dispersed in it. The mixture was heated at 70 °C for 8 h, during the course of which, it became a light yellow homogeneous solution. ^{1}H NMR indicated quantitative conversion of the components to Pd_{12}(**L1**)$_{24}$ cage assemblies. ^{1}H NMR (400 MHz, DMSO-d^6, 25 °C): δ 9.49 (bs, 96H), 8.38 (bm, 120H), 7.95 (bs, 48H), 5.40 (bs, 24H), 4.57 (bs, 48H). ^{13}C NMR (100 MHz, DMSO-d^6, 25 °C): δ 151.27(b), 149.46, 145.24, 135.01, 127.18(b), 124.34 (b), 62.18.

Pd_2(L2)$_4$ paddlewheel. To a standard NMR tube with **L2** (13.13 mg, 0.05006 mmol) dissolved in 366.7 μL of DMSO-d^6 was added via micropipette a solution of $Pd(NO_3)_2 \cdot 2H_2O$ (6.67 mg, 0.02503 mmol) in 133.3 μL of DMSO-d^6. The resultant mixture was agitated briefly to afford a light-yellow homogeneous solution; the head-space of the NMR tube was briefly purged with argon, and the NMR tube was sealed. The mixture in the NMR tube was heated at 70 °C for 8 h. ^{1}H NMR indicated quantitative conversion of the components to Pd_2(**L2**)$_4$ paddlewheel assemblies. ^{1}H NMR (400 MHz, DMSO-d^6, 25 °C): δ 10.22 (s, 8H), 9.45 (d, J = 5.6 Hz, 8H), 9.06 (s, 4H), 8.49 (d, J = 7.1 Hz, 8H), 7.91 (dd, J = 7.9, 5.9 Hz, 8H), 7.79 (s, 8H), 5.13 (b*), 4.55 (s, 8H). ^{13}C NMR (100 MHz, DMSO-d^6, 25 °C): δ 150.19, 149.23, 145.51, 138.51, 138.38, 134.98, 127.82, 126.02, 124.07, 62.38. FT-ICR-ESI HRMS: calcd. for $C_{36}H_{36}Br_4N_4$ [M – 3(NO_3^-)]$^{3+}$, most abundant m/z = 441.4132; found, 441.4141. *The resonance corresponding to the RO<u>H</u> proton is extremely broad and overlapping with nearby peaks, preventing accurate integration.

<u>Synthesis of **L2**, **L3**, telechelic PEG macromers **PL1** and **PL2**, and free ligand-substituted polyMOC gels</u>

***(3,5-di(pyridin-3-yl)phenyl)methanol* L2**. To a 100-mL round-bottom flask equipped with a magnetic stir-bar were added 3,5-dibromobenzyl alcohol (0.680 g, 2.56 mmol), pyridine-3-boronic acid pinacol ester (1.154 g, 5.63 mmol), potassium carbonate (3.536 g, 25.6 mmol), and tetrakis(triphenylphosphine)palladium (0.295 g, 0.255), and the vessel was sealed with a septum, evacuated and refilled with nitrogen three times. To the vessel via syringe were added anhydrous *N,N*-dimethylformamide (DMF, 20.7 mL) and then de-ionized water (0.430 mL, 23.9 mmol) which had first been sparged with argon for 30 min. The vessel was briefly evacuated until bubbling was observed, and then refilled with nitrogen, and briefly sparged with argon (10-15 sec). The vessel was heated in the oil bath for 60 h at 100 °C, with precipitated palladium black observed after 24 h. At the conclusion of the reaction, the vessel was brought to RT, and the contents of the vessel were filtered *in vacuo* over tightly-packed Celite® 545 on a medium-porosity glass frit, rinsing the flask and Celite with with MeOH (2 mL) and chloroform (3 x 70 mL). The filtrate was subjected to washes with water (4 x 100 mL and 1 x 500 mL), followed by drying over Na_2SO_4, filtration, and concentration *via* rotary evaporation. The remainder was subjected to chromatography on silica gel (CH_2Cl_2/MeOH step gradient: 10:0 → 9.5:0.5 → 9:1; **L2** eluted during the second step, R_f = 0.10 in CH_2Cl_2/MeOH = 9.5:0.5). The fractions containing the pure desired product were combined, concentrated by rotary evaporation, and dried *in vacuo* affording **L2** (0.3284 g, 48.9 % yield, ≥98.5% pure by LCMS) as a white to faint-yellow powdery solid. ^{1}H NMR (400 MHz, DMSO-d^6, 25 °C): δ 9.01 (dd, J = 2.4, 0.8 Hz, 2H), 8.60 (dd, J = 4.8, 1.6 Hz, 2H), 8.20 (ddd, J = 8.0, 2.4, 1.6 Hz, 2H), 7.93 (t, J = 1.7 Hz, 1H), 7.72

© 2015 Macmillan Publishers Limited. All rights reserved

(m, 2H), 7.52 (ddd, J = 8.0, 4.8, 0.8 Hz, 2H), 5.35 (t, J = 5.8 Hz, 1H), 4.67 (d, J = 5.8 Hz, 2H). ^{13}C NMR (100 MHz, DMSO-d^6, 25 °C): δ 148.67, 147.88, 144.51, 137.84, 135.44, 134.37, 124.67, 123.89, 123.85, 62.69. FT-ICR-DART HRMS: calcd. for $C_{17}H_{14}N_2O$ [M+H]$^+$, most abundant *m/z* = 263.1179; found, 263.1176.

***3,5-di(pyridin-4-yl)benzyl 4-(pyren-1-yl)butanoate* L3.** To a 1-dram scintillation vial with a magnetic stir-bar were added **L1** (0.0400 g, 0.153 mmol), 4-(1-pyrenyl)butyric acid (0.0440 g, 0.153 mmol), *N*-(3-dimethylaminopropyl)-*N'*-ethylcarbodiimide hydrochloride (EDC·HCl) (0.0380 g, 0.198 mmol), and 4-(dimethylamino)pyridine (DMAP) (0.0048g, 0.040 mmol). The vial was brought into the glove box, and to it was added dichloromethane (DCM, 0.5 mL), and the reaction mixture was sealed and allowed to stir for 24 h. The reaction mixture was then removed from the glove box and loaded directly onto silica gel packed inside a 2-mL Pasteur pipette for column chromatography, using ethyl acetate as the eluent (R_f of **L3** = 0.10). The fractions containing **L3** were combined, concentrated via rotary evaporation, and subjected to column chromatography using ethyl acetate as the eluent. Rotary evaporation of the combined fractions containing the pure product, followed by drying at 60 °C overnight on the Schlenk line afforded **L3** (0.0687 g, 84.6 %) as a beige-yellow powdery solid. ^{1}H NMR (400 MHz, DCM-d^2, 25 °C): δ 8.66 (dd, J = 4.6, 1.6 Hz, 4H), 8.29 (d, J = 9.3 Hz, 1H), 8.17 (dd, J = 7.6, 1.1, Hz, 1H), 8.14 (dd, J = 7.7, 1.0 Hz, 1H), 8.10 (d, J = 7.8 Hz, 1H), 8.05 (d, J = 9.3 Hz, 1H), 8.03 (s, 2H), 7.99 (t, J = 7.6 Hz, 1H), 7.88—7.83 (m, 2H), 7.72 (d, J = 1.7 Hz, 2H), 7.60 (bd, J = 6.0 Hz, 4H), 5.25 (s, 2H), 3.41 (t, J = 7.6 Hz, 2H), 2.56 (t, J = 7.3 Hz, 2H), 2.23 (tt, J = 7.6, 7.3 Hz, 2H). ^{13}C NMR (100 MHz, DCM-d^2, 25 °C): δ 173.41 (ester carbon), 150.64, 147.80, 139.92, 138.79, 136.25, 131.79, 131.25, 130.35, 129.12, 127.82, 127.78, 127.69, 127.66, 127.07, 126.31, 125.90, 125.37, 125.33, 125.24, 125.19, 125.15, 123.69, 122.08, 65.95, 34.18, 33.01, 27.19. FT-ICR-DART HRMS: calcd. for $C_{37}H_{28}N_2O_2$ [M+H]$^+$, most abundant *m/z* = 533.2224; found, 533.2216.

PL1. To a 20-mL microwave vial equipped with a magnetic stir-bar were added *finely powdered* **L1** (0.300 g, 1.14 mmol), PEG-diacid (M_n = 2.2 kDa, 0.7741 g, 0.35 mmol), EDC·HCl (0.4336 g, 2.26 mmol), and DMAP (0.0870 g, 0.712), and the vessel was brought into the glove box. To the vessel was added DCM (3.84 mL), ensuring that the solid was washed down the walls of the vial, and the vial was crimped and set to stir at RT. During the first several minutes, most of the solid dissolved, giving rise to a yellow solution. After 25 h, the vessel was removed from the glove box and concentrated *via* rotary evaporation. The product was extracted from the oily mixture with toluene (3 x 2 mL), filtering the extracts through Celite® 545 packed in Pasteur pipettes. The combined extracts were concentrated by rotary evaporation, redissolved in 2 mL toluene, and precipitated into cold (~ -10 – -20 °C) diethyl ether (35 mL). The vial containing the solution of crude product was rinsed with an additional 0.5 mL toluene, and this rinsing was also subjected to precipitation. The precipitation was allowed to proceed overnight at -20 °C, and the precipitated product was collected by filtration *in vacuo*, rinsing with cold diethyl ether (2 x 40 mL, 1 x 100 mL). Quickly, while still cold, the white solid was transferred from the filter disk to a 20-mL scintillation vial and was dried *in vacuo* overnight. The dry solid (~950 mg) was redissolved in water (9.5 mL, 18.2 MΩcm at RT, from Milli-Q® system), filtered through a nylon syringe filter with 0.45 μm-pore size, and subjected to prep-HPLC purification. The combined pure fractions were diluted with sat. $NaHCO_{3(aq.)}$ until pH was ~7–8. The aqueous phase was divided into two portions and the product was extracted from each one with DCM (5 x

© 2015 Macmillan Publishers Limited. All rights reserved

300 mL). The combined extracts were dried over anhydrous Na_2SO_4, concentrated by rotary evaporation, re-dissolved in 1 mL DCM, and precipitated into cold diethyl ether (35 mL). The vial containing the dissolved pure product was rinsed with an additional 0.5 mL DCM, and this rinsing was also subjected to precipitation. The precipitation was allowed to proceed overnight at -20 °C, and the precipitated product was collected by filtration *in vacuo*, rinsing with cold diethyl ether (2 x 40 mL, 1 x 100 mL). Quickly, while still cold, the white solid was transferred from the filter disk to a 20-mL scintillation vial and was dried *in vacuo* overnight, affording **PL1** (0.3752 g, 40 % yield) as a soft white solid. ^{1}H NMR (400 MHz, CD_2Cl_2, 25 °C): δ 8.69 (bdd, J = 4.3, 1.4 Hz, 8H), 7.88 (t, J = 1.7 Hz, 2H), 7.73 (d, J = 1.7 Hz, 4H), 7.59 (dd, J = 4.5, 1.7 Hz, 8H), 5.33 (s, 4H), 4.22 (s, 4H), 3.80—3.37 (m, 204H). ^{13}C NMR (100 MHz, CD_2Cl_2, 25 °C): δ 170.55, 150.77, 147.64, 140.04, 138.23, 127.83, 126.09, 122.07, 71.33, 70.90 (b), 70.86, 68.98, 66.19. MALDI-TOF: M_n = 2.7 kDa; calcd. for $C_{132}H_{218}N_4O_{52}$ $[M+H]^+$, most abundant *m/z* = 2693.5; found, 2693.6. GPC (THF, 25 °C): M_w = 2.83 kDa, M_n = 2.73 kDa (obtained via conversion from values determined via calibration with polystyrene standards); $Đ$ = 1.03. M_n (^{1}H NMR) = 2.87 kDa. M_n = 2.7 kDa was employed in the calculations for reactions involving **PL1**.

PL2. **PL2** was prepared identically to **PL1**, except using **L2** instead of **L1**. **PL2** was isolated as a soft white solid (0.4208 g, 45 % yield). ^{1}H NMR (400 MHz, CD_2Cl_2, 25 °C): δ 8.90 (dd, J = 2.3, 0.5 Hz, 4H), 8.62 (dd, J = 4.8, 1.6 Hz, 4H), 7.97 (ddd, J = 7.9, 2.4, 1.7 Hz, 4H), 7.79 (t, J = 1.7 Hz, 2H), 7.65 (d, J = 1.7 Hz, 4H), 7.42 (ddd, J = 7.9, 4.8, 0.8 Hz, 4H), 5.33 (s, 4H), 4.22 (s, 4H), 3.81—3.36 (m, 194H). ^{13}C NMR (100 MHz, CD_2Cl_2, 25 °C): δ 170.56, 149.34, 148.64, 139.64, 138.08, 136.16, 134.83, 127.01, 126.32, 124.03, 71.32, 70.90 (b), 70.85, 68.98, 66.32. MALDI-TOF: M_n = 2.7 kDa; calcd. for $C_{132}H_{218}N_4O_{52}$ $[M+H]^+$, most abundant *m/z* = 2693.5; found, 2693.8. GPC (THF, 25 °C): M_w = 2.81 kDa, M_n = 2.74 kDa (obtained via conversion from values determined via calibration with polystyrene standards); $Đ$ = 1.03. M_n (^{1}H NMR) = 2.76 kDa. M_n = 2.7 kDa was employed in the calculations for reactions involving **PL2**.

PolyMOC gels with macromer replaced with free ligand. General synthesis: The procedure is identical to that used for the synthesis of regular polyMOC gels, except during the preparation of the macromer solution, *x*% of the macromer was replaced with 2 equivalents of the corresponding *free* ligand to achieve the same total concentration of dipyridine ligands (e.g., when *x*% = 25%, instead of 20.25 mg of macromer **PL1**, 15.18 mg of **PL1** (0.56 μmol) was combined with 0.98 mg of **L1** (0.38 μmol) in 210.0 μL DMSO-d^6).

Supplementary References

S1. Mori, S. Calibration of size exclusion chromatography columns for determination of polymer molecular weight distribution. *Analytical Chemistry* **53**, 1813-1818 (1981).
S2. Fulmer, G. R. *et al.* NMR Chemical Shifts of Trace Impurities: Common Laboratory Solvents, Organics, and Gases in Deuterated Solvents Relevant to the Organometallic Chemist. *Organometallics* **29**, 2176-2179 (2010).
S3. Kienberger, F. *et al.* Static and Dynamical Properties of Single Poly(Ethylene Glycol) Molecules Investigated by Force Spectroscopy. *Single Molecules* **1**, 123-128 (2000).
S4. Sheldrick, G. Phase annealing in SHELX-90: direct methods for larger structures. *Acta Crystallogr., Sect. A: Fundam. Crystallogr.* **46**, 467-473 (1990).

© 2015 Macmillan Publishers Limited. All rights reserved

S5. Sheldrick, G. A short history of SHELX. *Acta Crystallogr., Sect. A: Fundam. Crystallogr.* **64**, 112-122 (2008).
S6. Müller, P. Practical suggestions for better crystal structures. *Crystallogr. Rev.* **15**, 57-83 (2009).
S7. Kline, S. R. *Journal of Applied Crystallography* **2006**, *39*, 895–900.
S8. Hore, M. J. A.; Ford, J.; Ohno, K.; Composto, R. J.; Hammouda, B. *Macromolecules* **2013**, *46*, 9341–9348.
S9. Hammouda, B. *Advances in Polymer Science* **1993**, *106*, 87-133.
S10. Guth, E. & James, H. M. Elastic and Thermoelastic Properties of Rubber like Materials. *Ind. Eng.Chem.* **33**, 624-629, (1941).
S11. Rubinstein, M. & Colby, R. *Polymers Physics*. (Oxford, 2003).
S12. Tominaga, M. *et al.* Finite, spherical coordination networks that self-organize from 36 small components. *Angew. Chem., Int. Ed.* **43**, 5621-5625 (2004).
S13. Harris, J. M., Kozlowski, A. & Guo, L. Method of preparing carboxylic acid functionalized polymers. US Patent 8,067,505 filed 9 Feb., and issued 29 Nov. 2011.

Supplementary Figure S1

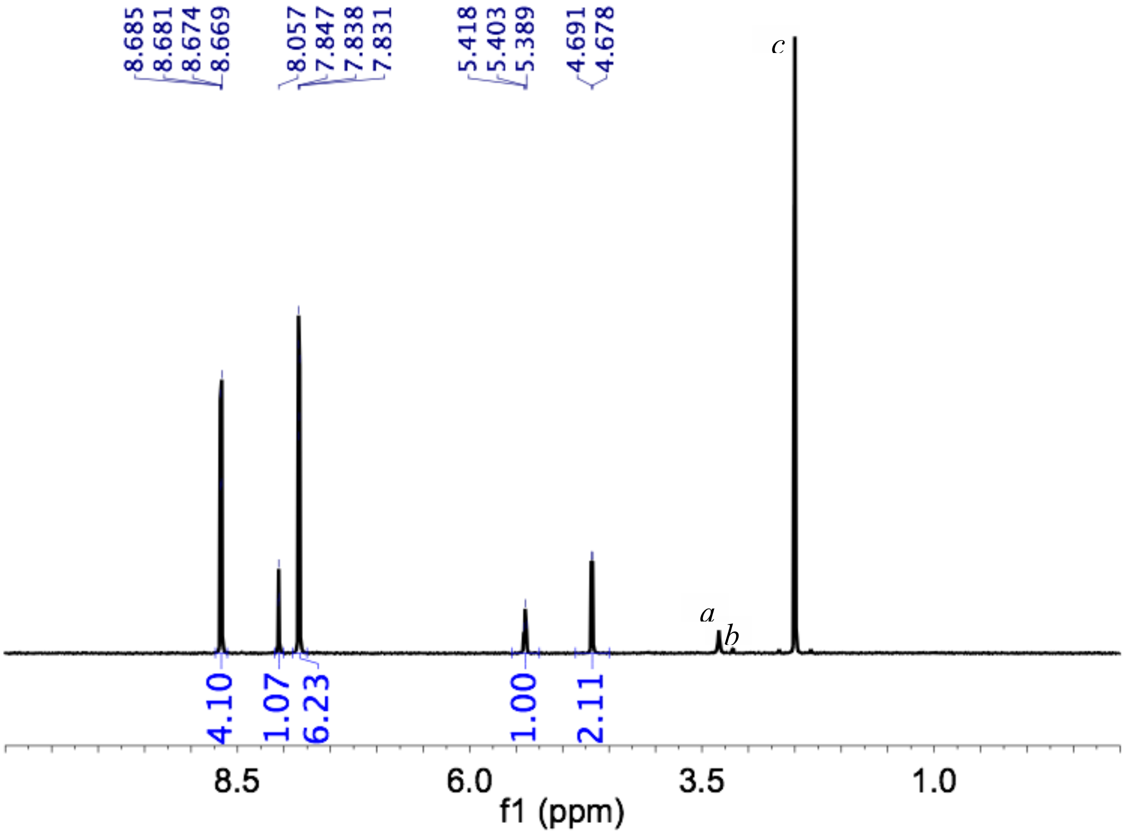

^{1}H NMR of **L1** at 25 °C in DMSO-d^6. $^{a, b, c}$These resonances are due to water (*a*), methanol (b, ~3%) and residual DMSO-d^5 (*c*).

© 2015 Macmillan Publishers Limited. All rights reserved

Supplementary Figure S2

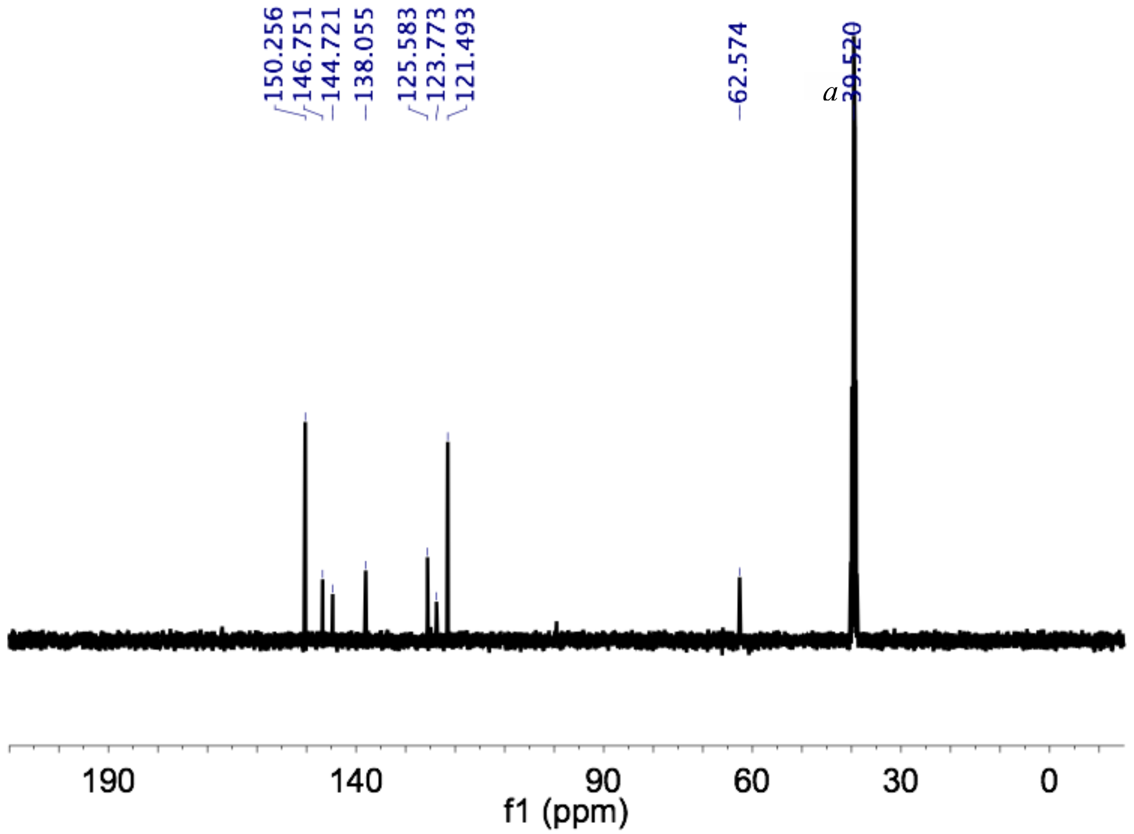

^{13}C NMR of **L1** at 25 °C in DMSO-d^6. [a]This resonance is due to DMSO-d^6.

© 2015 Macmillan Publishers Limited. All rights reserved

Supplementary Figure S3

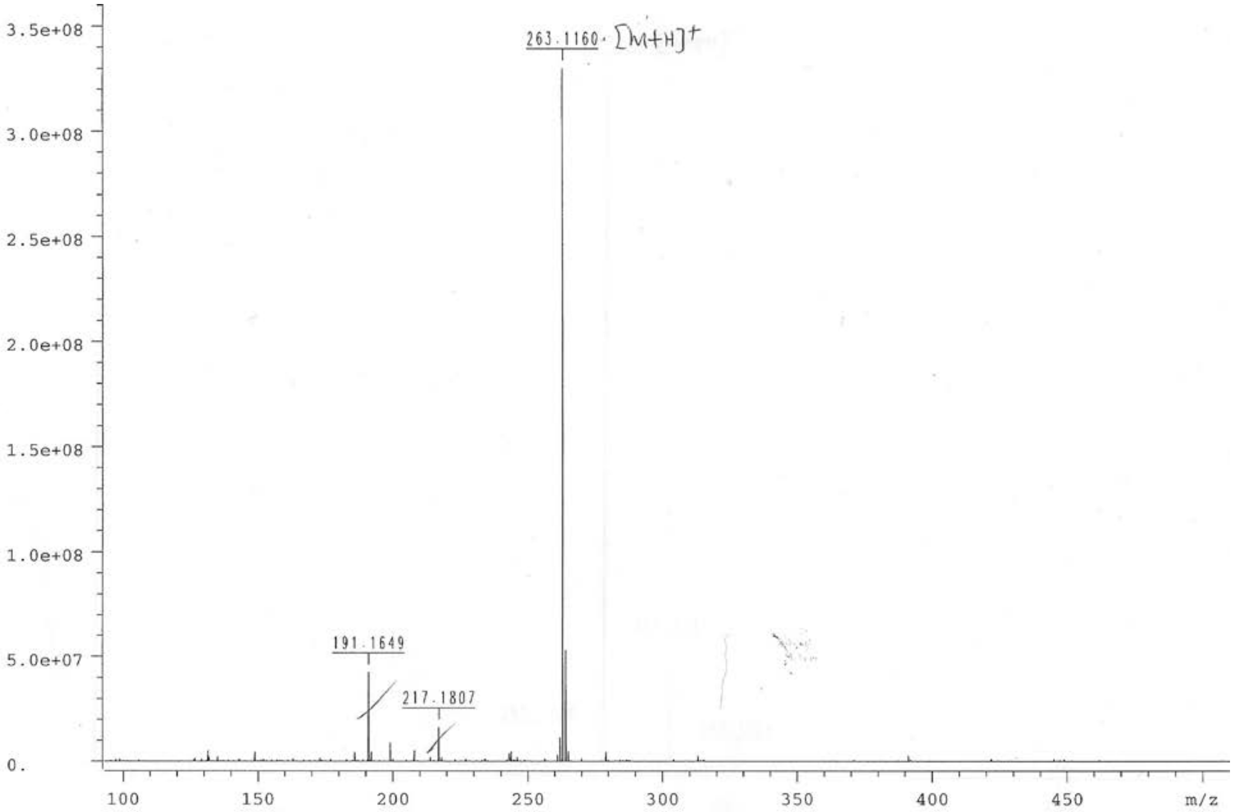

FT-ICR-ESI mass spectrum of **L1**. Peaks marked with slashes correspond to the background signals.

© 2015 Macmillan Publishers Limited. All rights reserved

Supplementary Figure S4

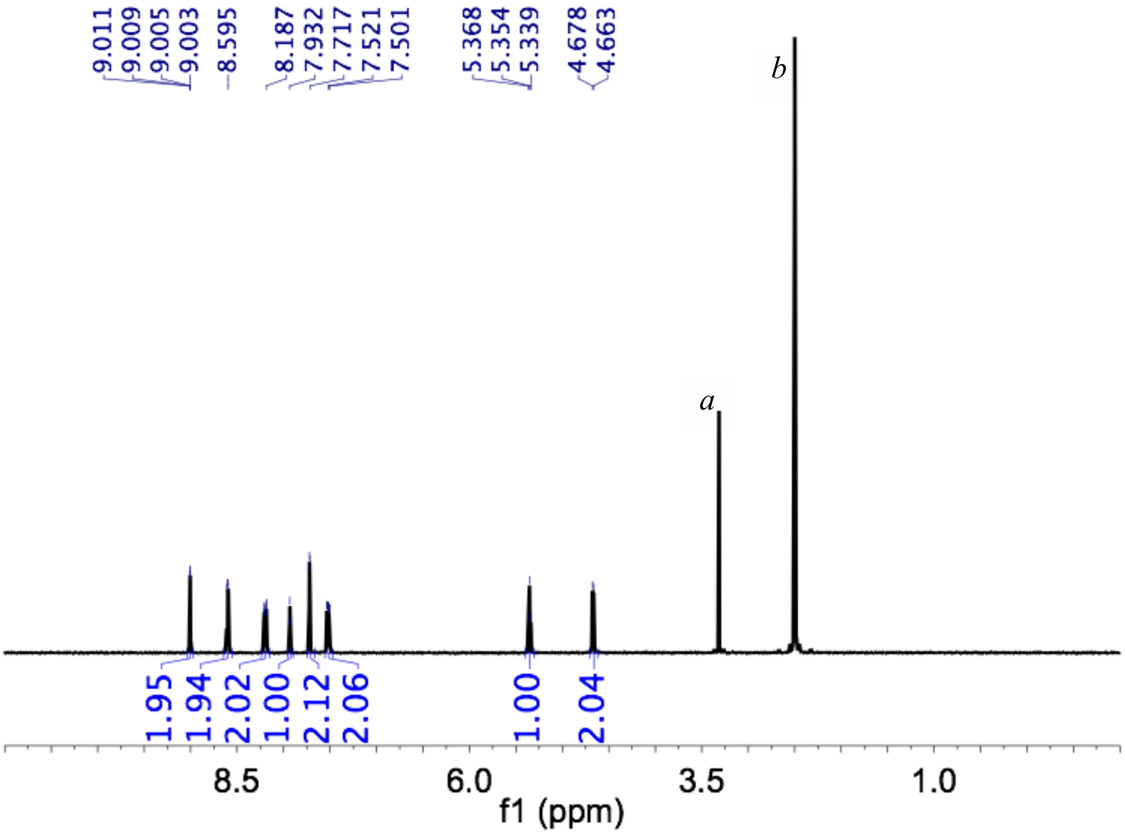

^{1}H NMR of **L2** at 25 °C in DMSO-d^6. $^{a,\,b}$These resonances are due to water (*a*) and residual DMSO-d^5 (b).

© 2015 Macmillan Publishers Limited. All rights reserved

Supplementary Figure S5

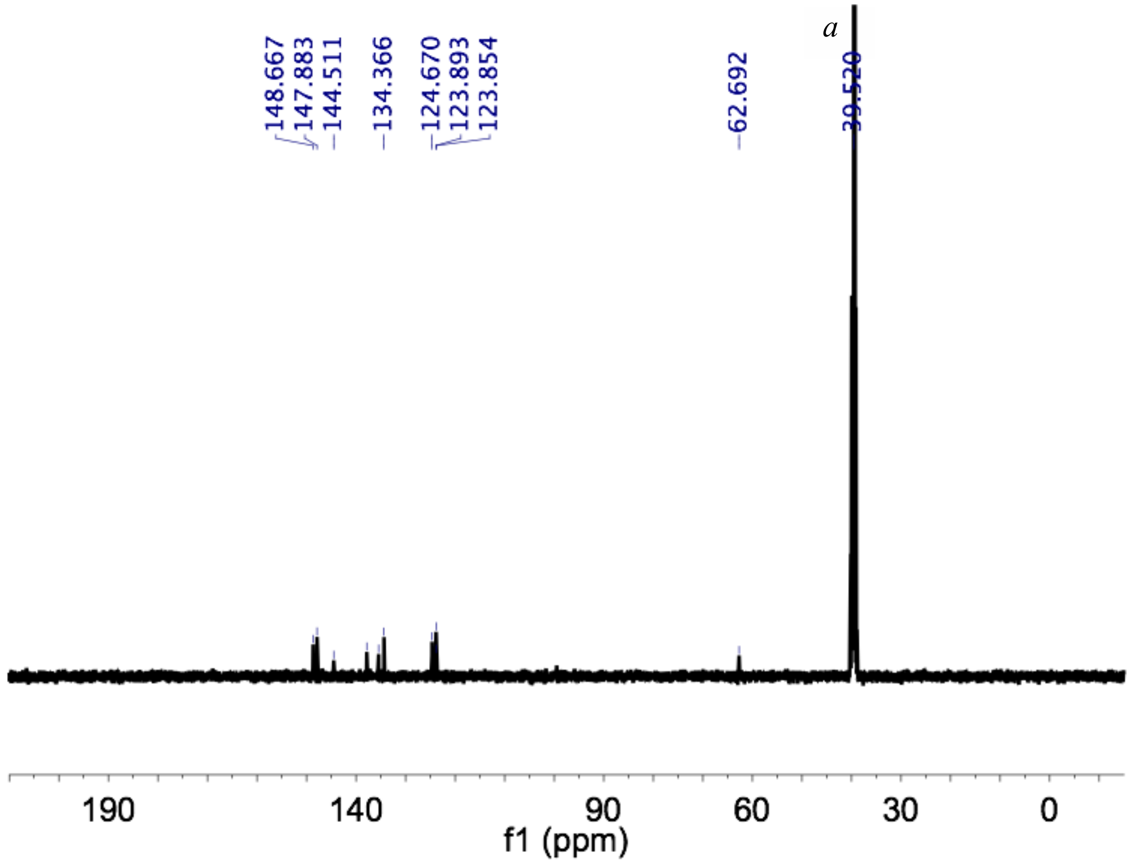

^{13}C NMR of **L2** at 25 °C in DMSO-d^6. [a]This resonance is due to DMSO-d^6.

© 2015 Macmillan Publishers Limited. All rights reserved

Supplementary Figure S6

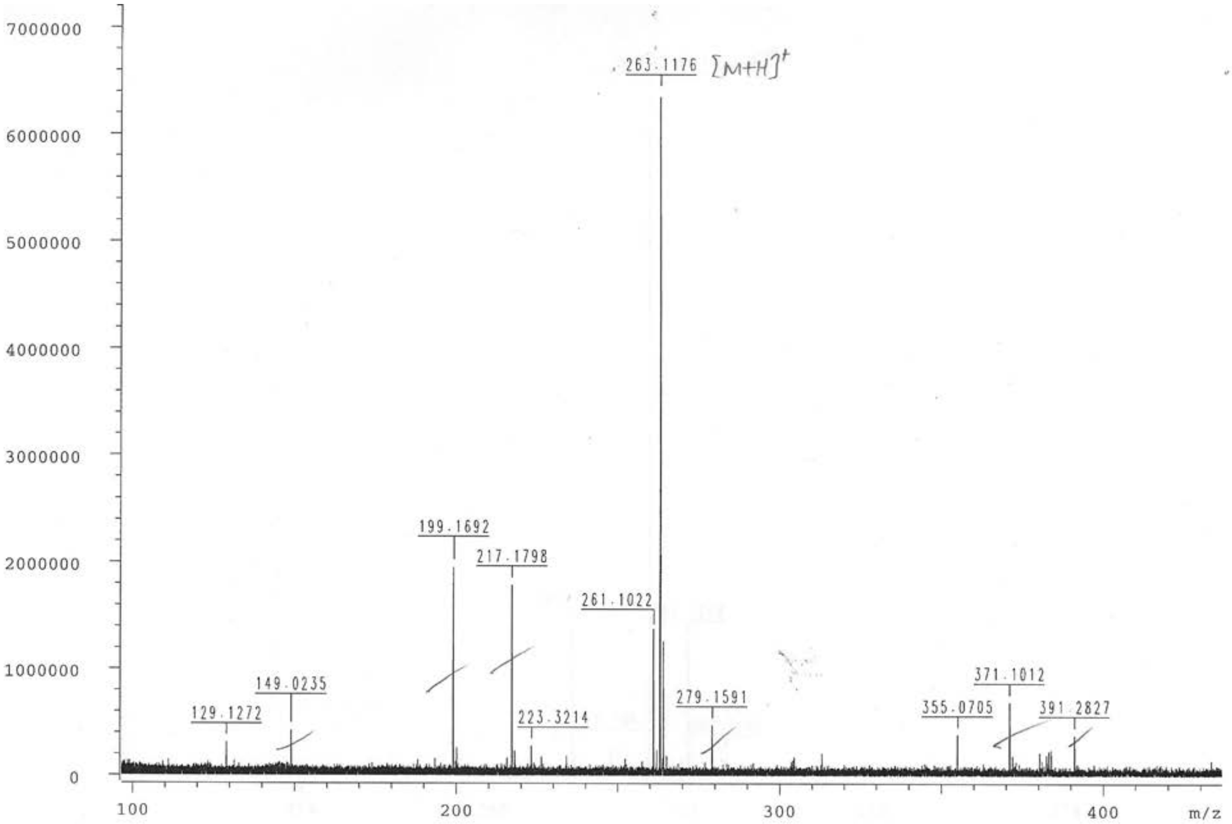

FT-ICR-ESI mass spectrum of **L2**. Peaks marked with slashes correspond to the background signals.

© 2015 Macmillan Publishers Limited. All rights reserved

Supplementary Figure S7

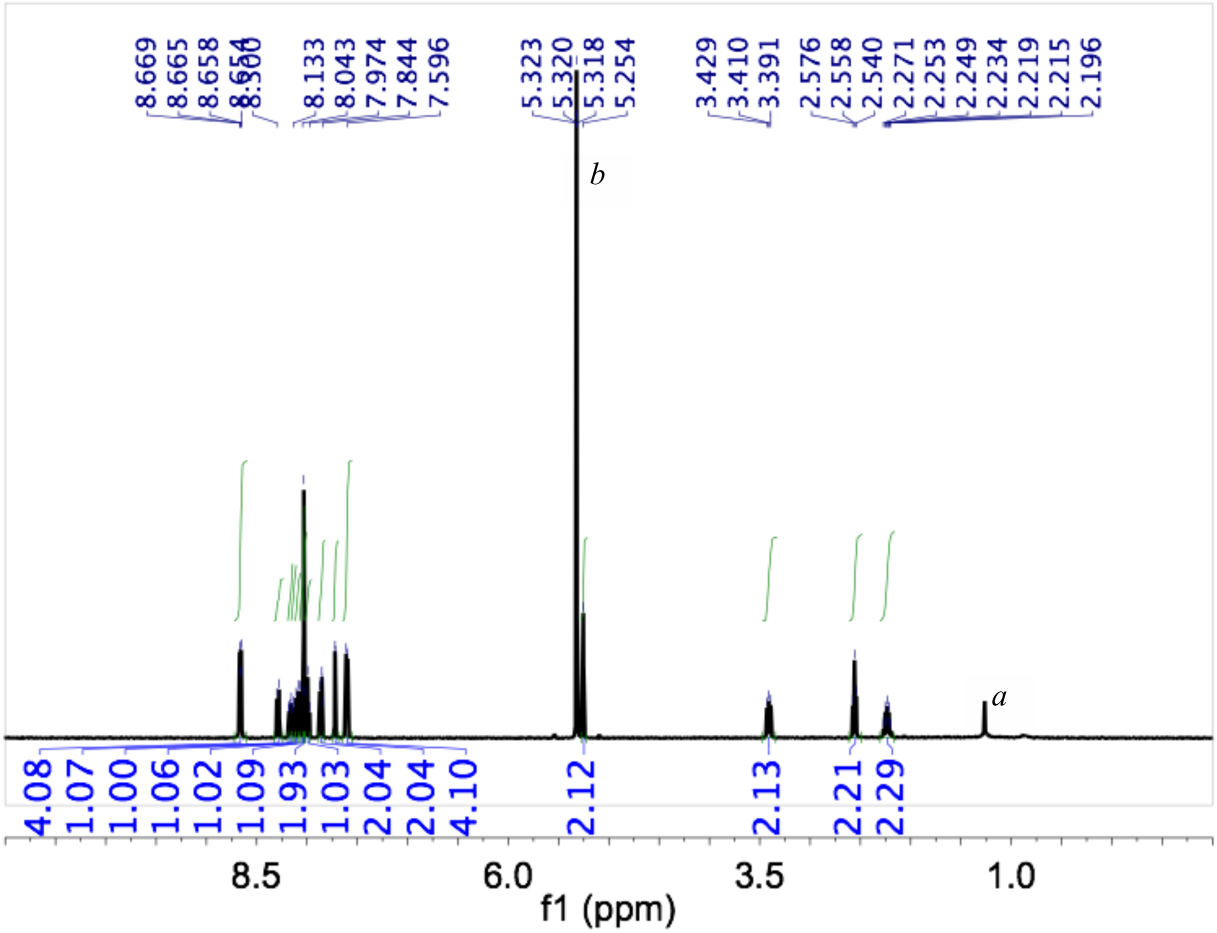

^{1}H NMR of **L3** at 25 °C in DCM-*d*[2]. [a, b]These resonances are due to H grease (*a*) and residual DCM-*d*[1] (*b*).

© 2015 Macmillan Publishers Limited. All rights reserved

Supplementary Figure S8

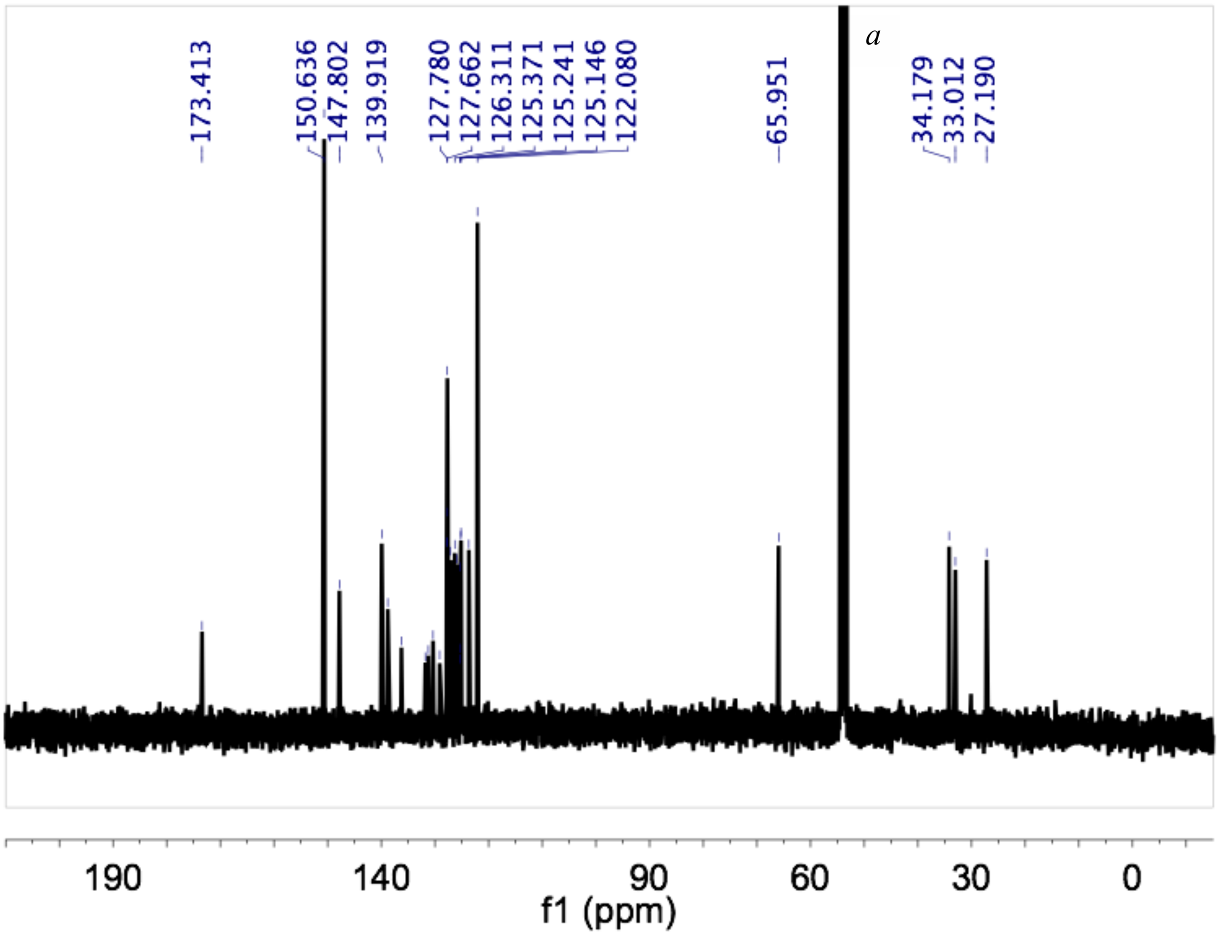

^{13}C NMR of **L3** at 25 °C in DCM-d^2. [a]This resonance is due to DCM-d^2 (*a*).

© 2015 Macmillan Publishers Limited. All rights reserved

Supplementary Figure S9

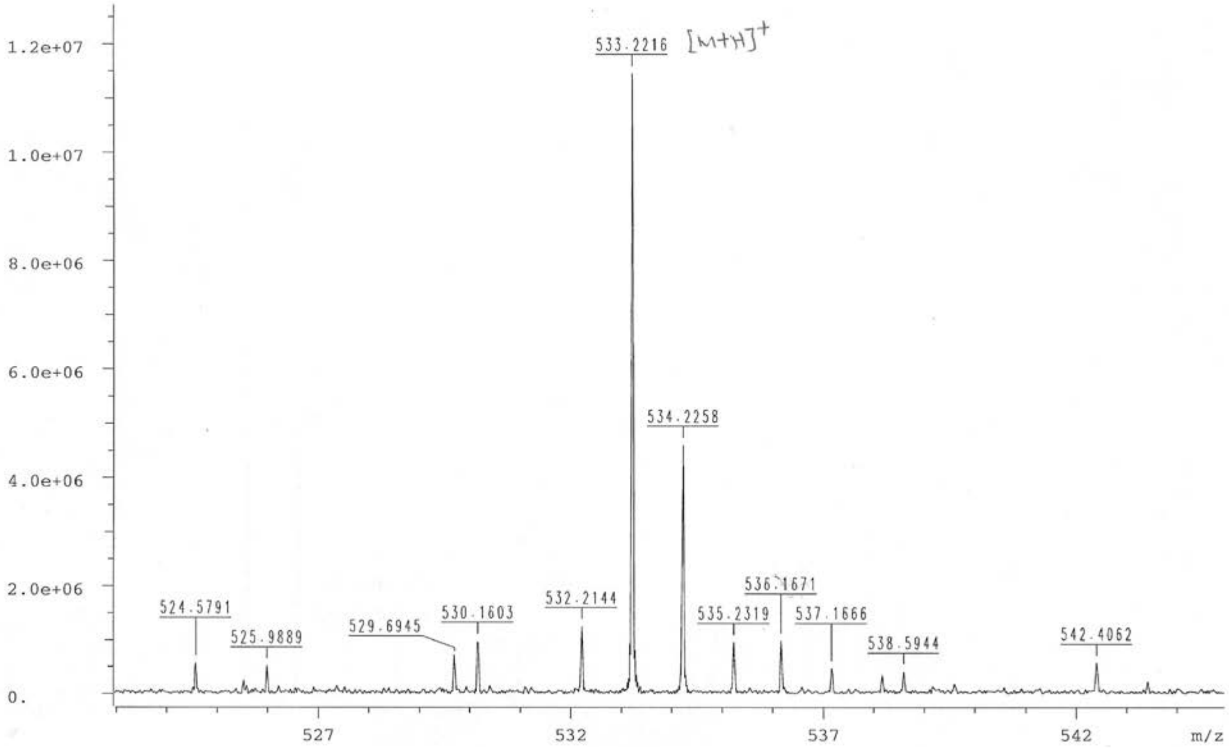

A portion of the FT-ICR-DART mass spectrum of **L3** showing the isotope distribution of the parent ion of **L3** $[M+H]^+$.

© 2015 Macmillan Publishers Limited. All rights reserved

Supplementary Figure S10

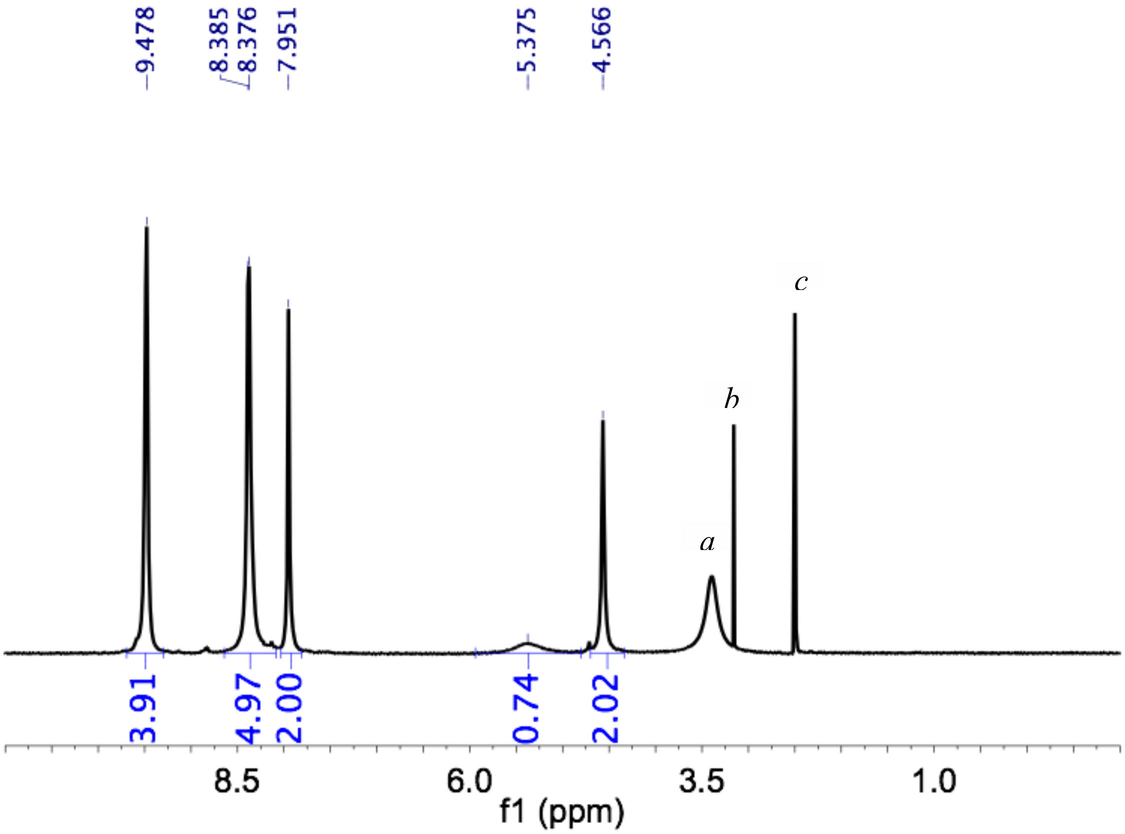

^{1}H NMR of Pd_{12}(**L1**)$_{24}$ cage assembly at 25 °C in DMSO-d^6. [a, b,c] These resonances are due to water (*a*), methanol (b, 2% per **L1**) and residual DMSO-d^5 (*c*).

© 2015 Macmillan Publishers Limited. All rights reserved

Supplementary Figure S11

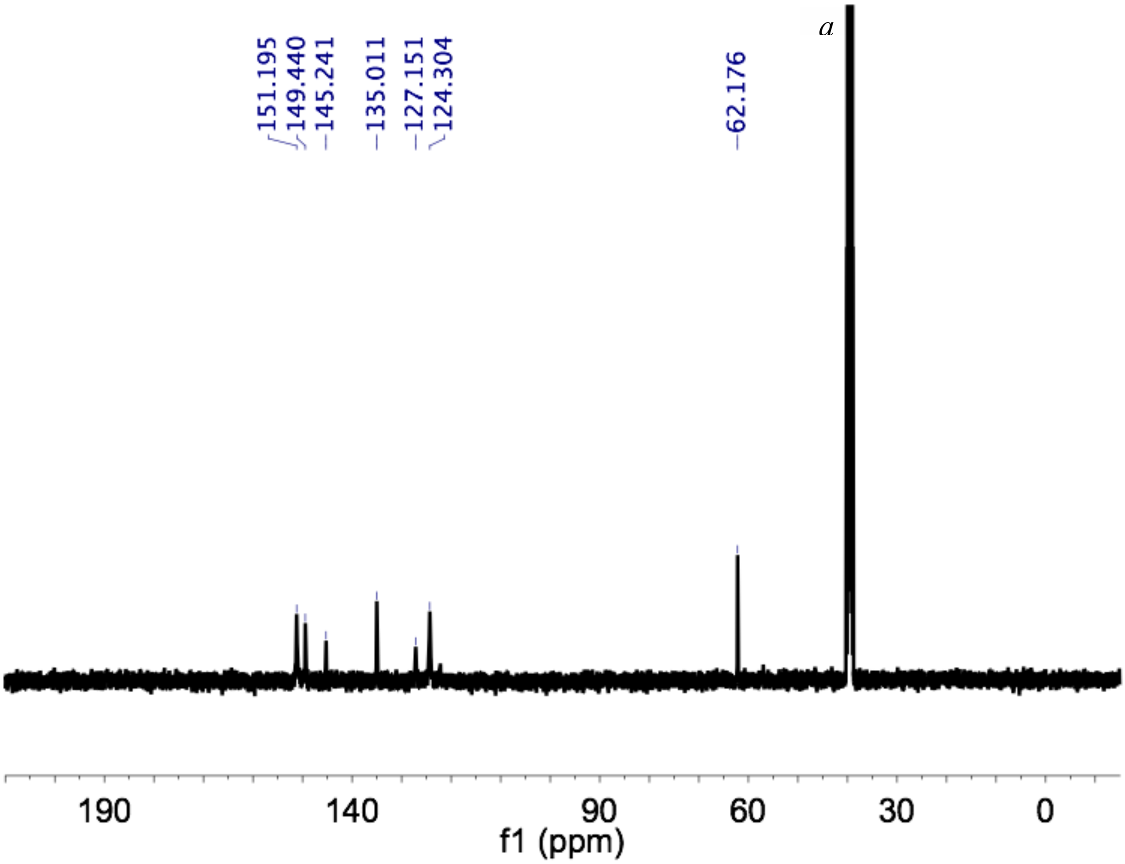

^{13}C NMR of Pd_{12}(**L1**)$_{24}$ cage assembly at 25 °C in DMSO-d^6. ^{a}This resonance is due to DMSO-d^6.

© 2015 Macmillan Publishers Limited. All rights reserved

Supplementary Figure S12

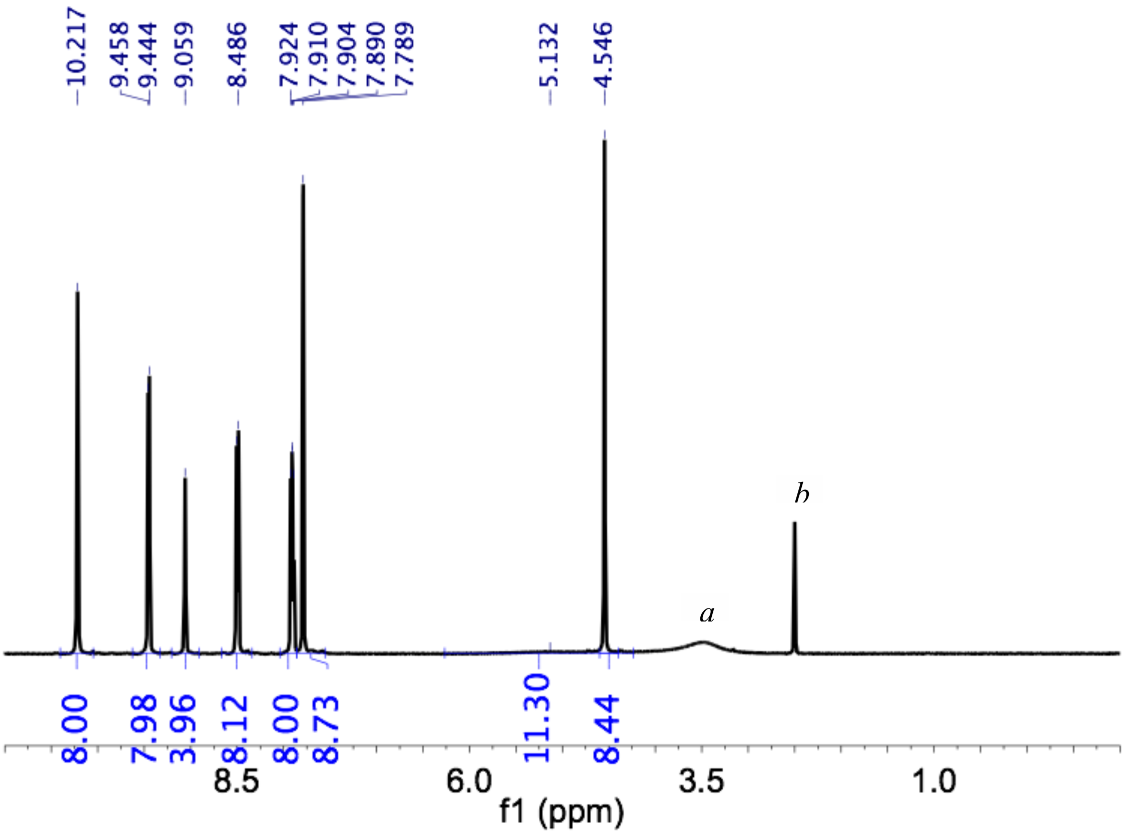

^{1}H NMR of Pd_2(**L2**)$_4$ paddlewheel assembly at 25 °C in DMSO-d^6. [a, b]These resonances are due to water (*a*) and residual DMSO-d^5 (*b*).

© 2015 Macmillan Publishers Limited. All rights reserved

DOI: 10.1038/NCHEM.2390

Supplementary Figure S13

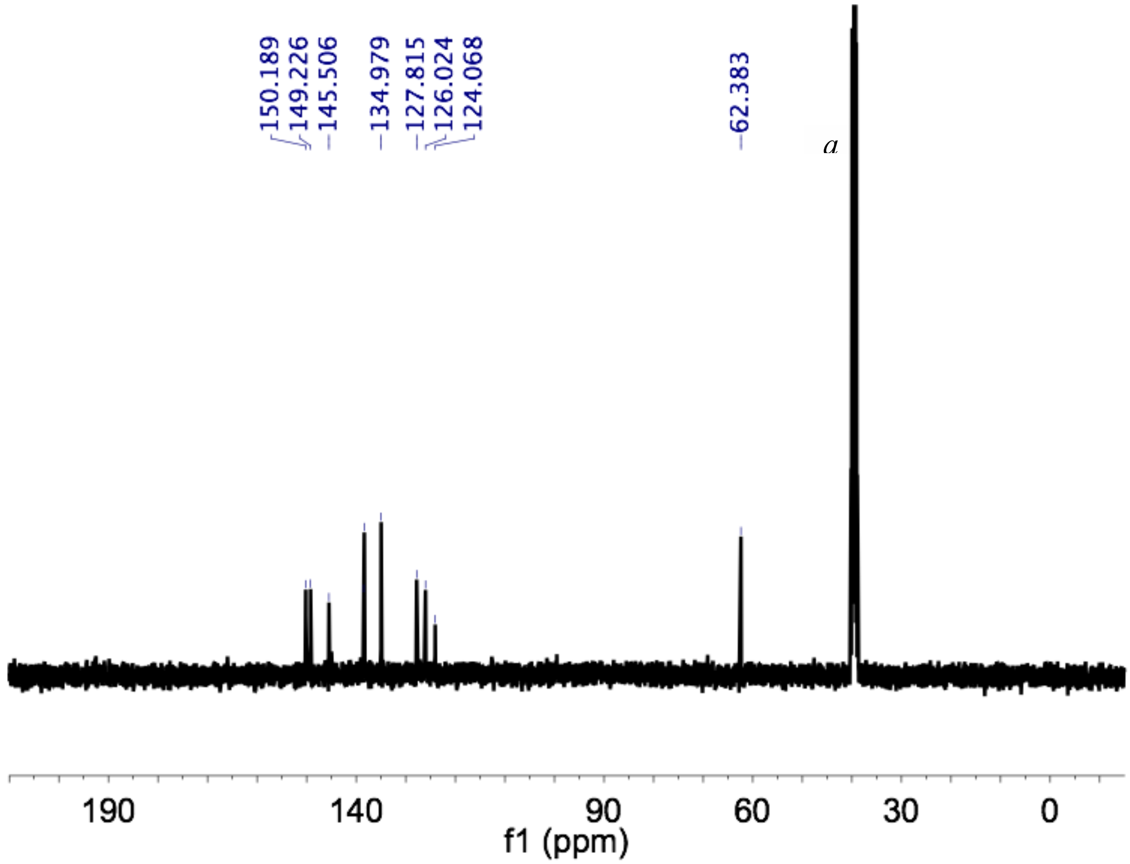

^{13}C NMR of Pd_2(**L2**)$_4$ paddlewheel assembly at 25 °C in DMSO-d^6. [a]This resonance is due to DMSO-d^6.

© 2015 Macmillan Publishers Limited. All rights reserved

Supplementary Figure S14

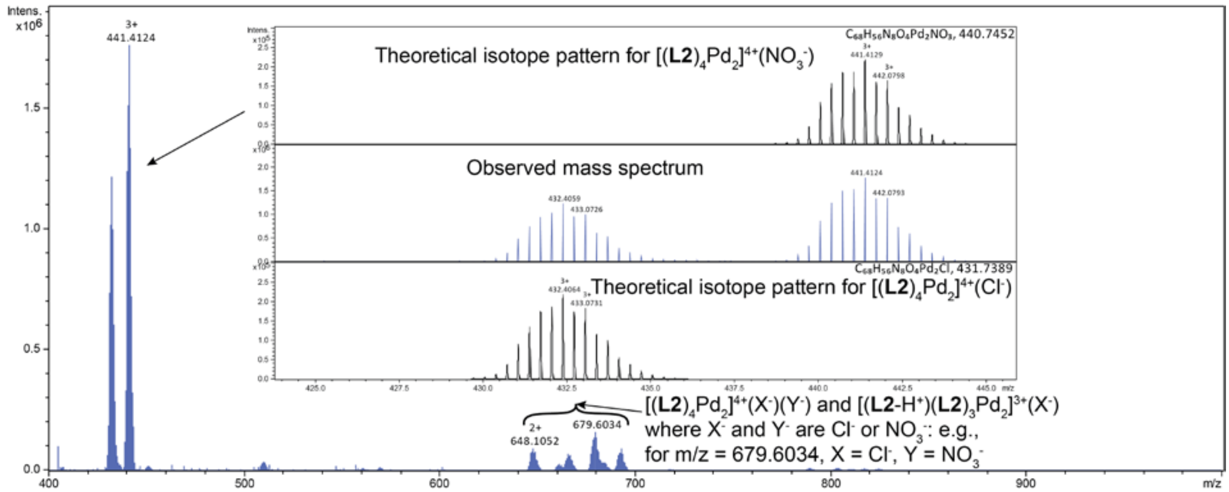

High resolution ESI-TOF mass spectrum of the $Pd_2($**L2**$)_4$ paddlewheel assembly.

© 2015 Macmillan Publishers Limited. All rights reserved

Supplementary Figure S15

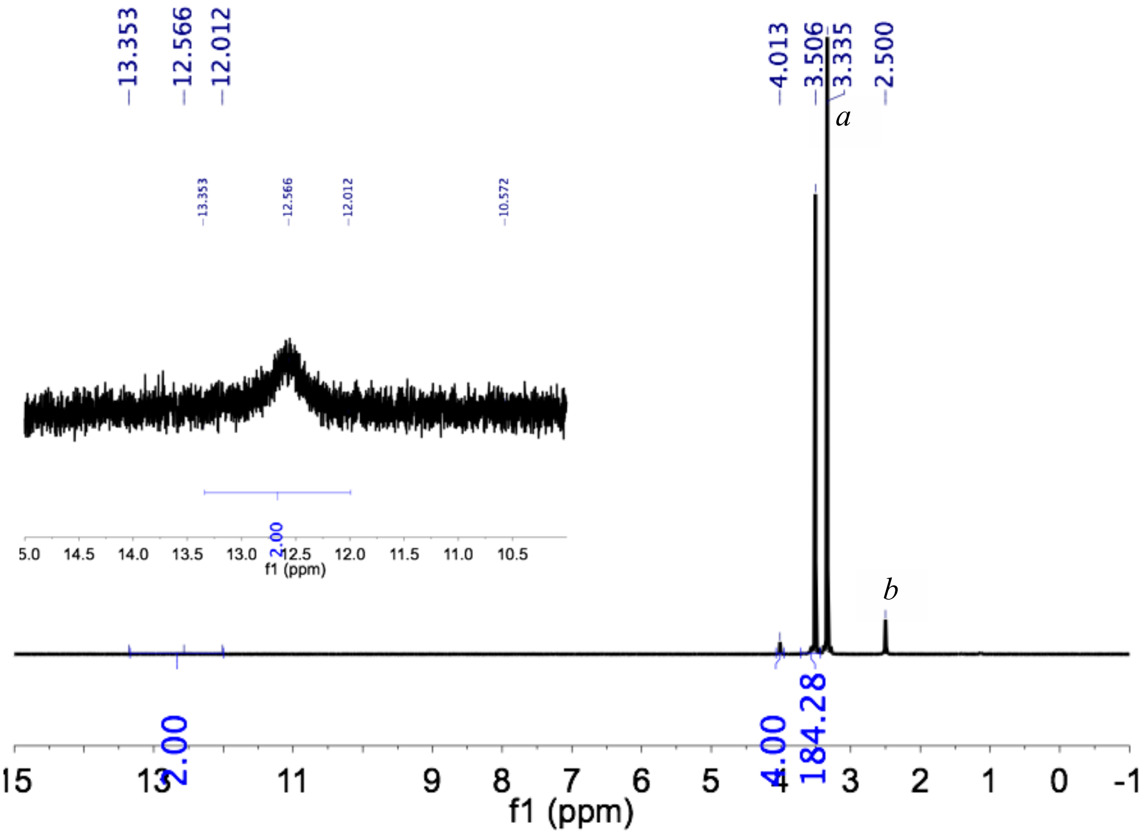

^{1}H NMR of **PEG-diacid** (M_n=2.2 kDa) at 25 °C in DMSO-d^6. [a, b]These resonances are due to water (*a*) and residual DMSO-d^5 (*b*). Inset is the zoomed-in region corresponding to the carboxylic acid proton resonance.

© 2015 Macmillan Publishers Limited. All rights reserved

Supplementary Figure S16

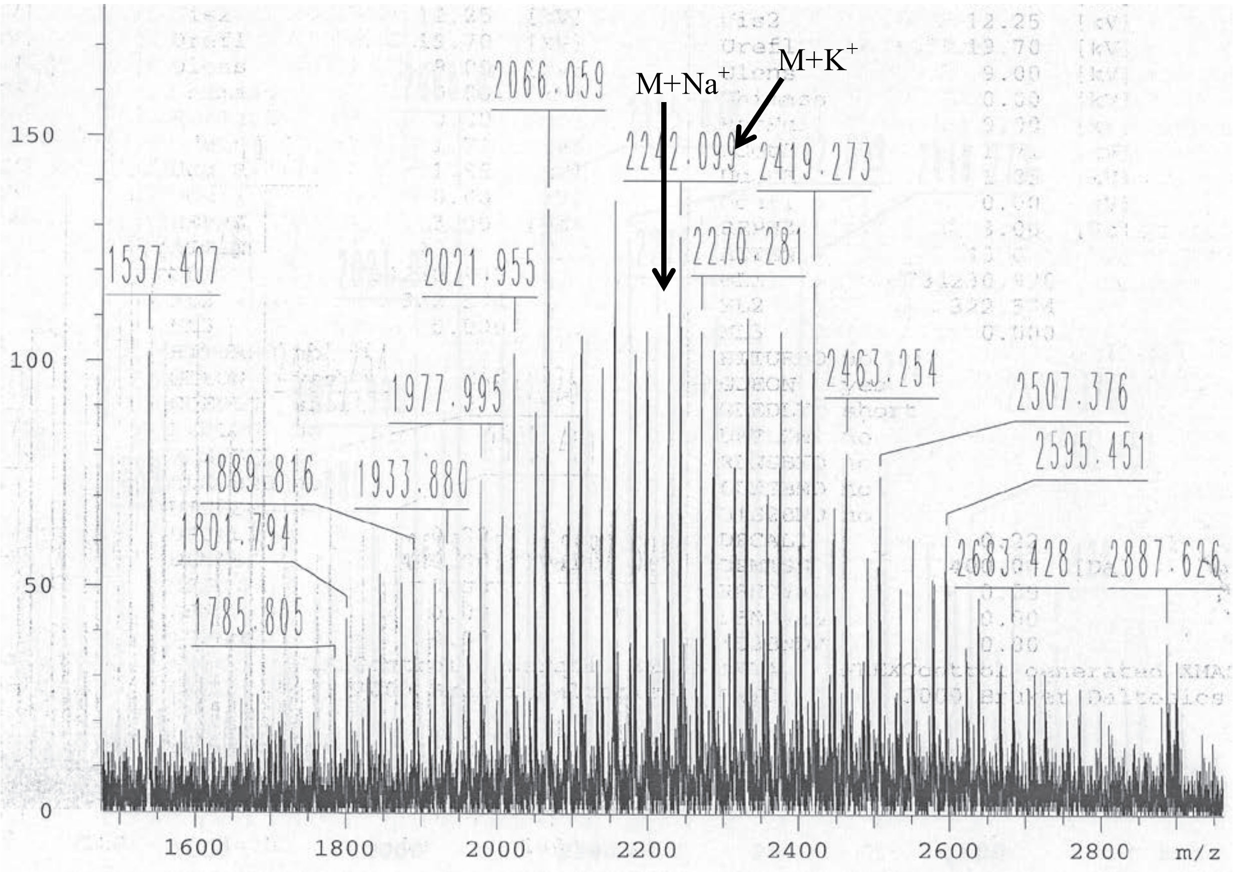

MALDI-TOF mass spectrum of **PEG-diacid** (M_n = 2.2 kDa).

© 2015 Macmillan Publishers Limited. All rights reserved

Supplementary Figure S17

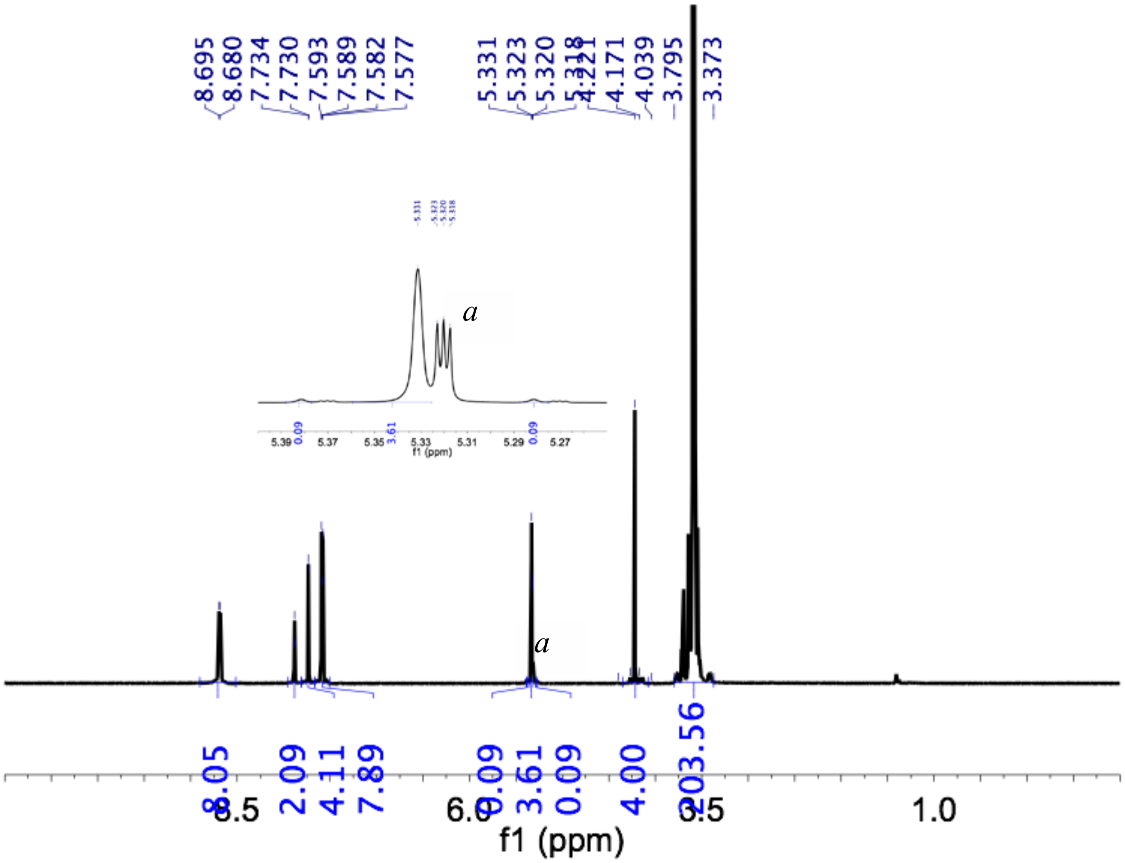

^{1}H NMR of **PL1** at 25 °C in CD_2Cl_2. [a] This resonance is due to residual $CHDCl_2$ (see inset).

© 2015 Macmillan Publishers Limited. All rights reserved

Supplementary Figure S18

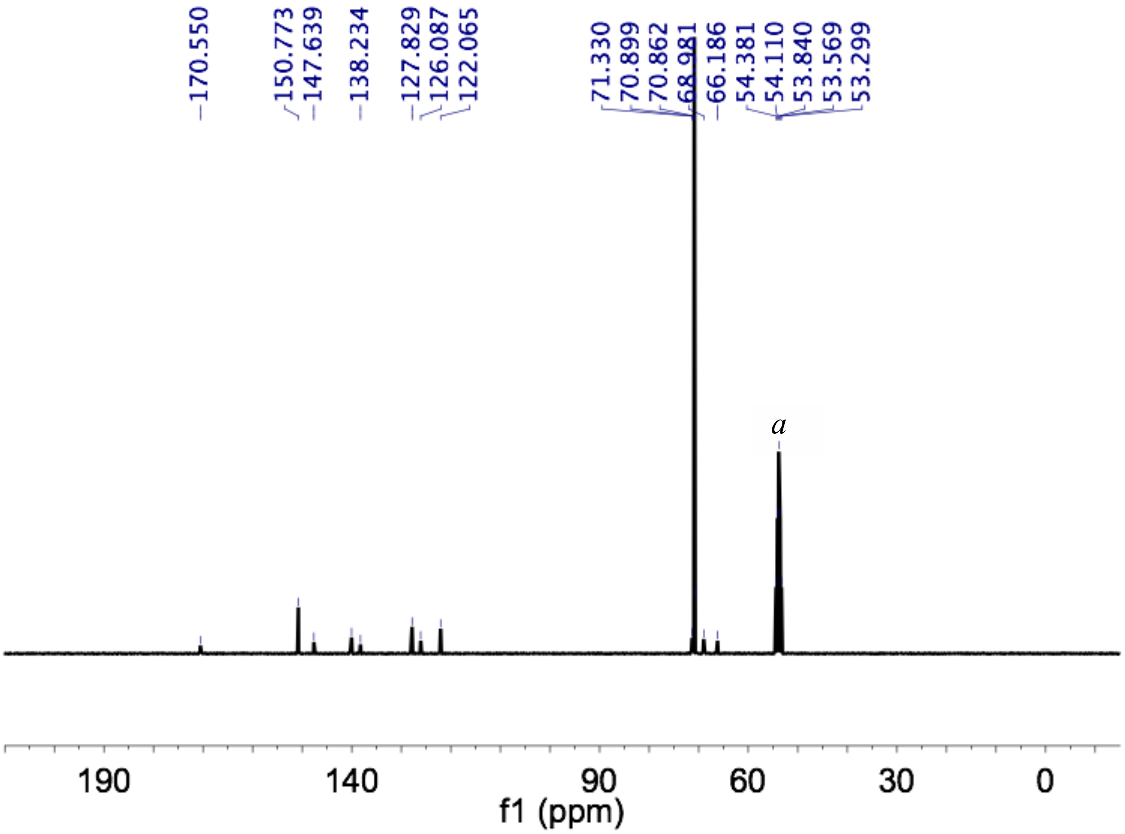

^{13}C NMR of **PL1** at 25 °C in CD_2Cl_2. [a] This resonance is due to CD_2Cl_2.

© 2015 Macmillan Publishers Limited. All rights reserved

Supplementary Figure S19

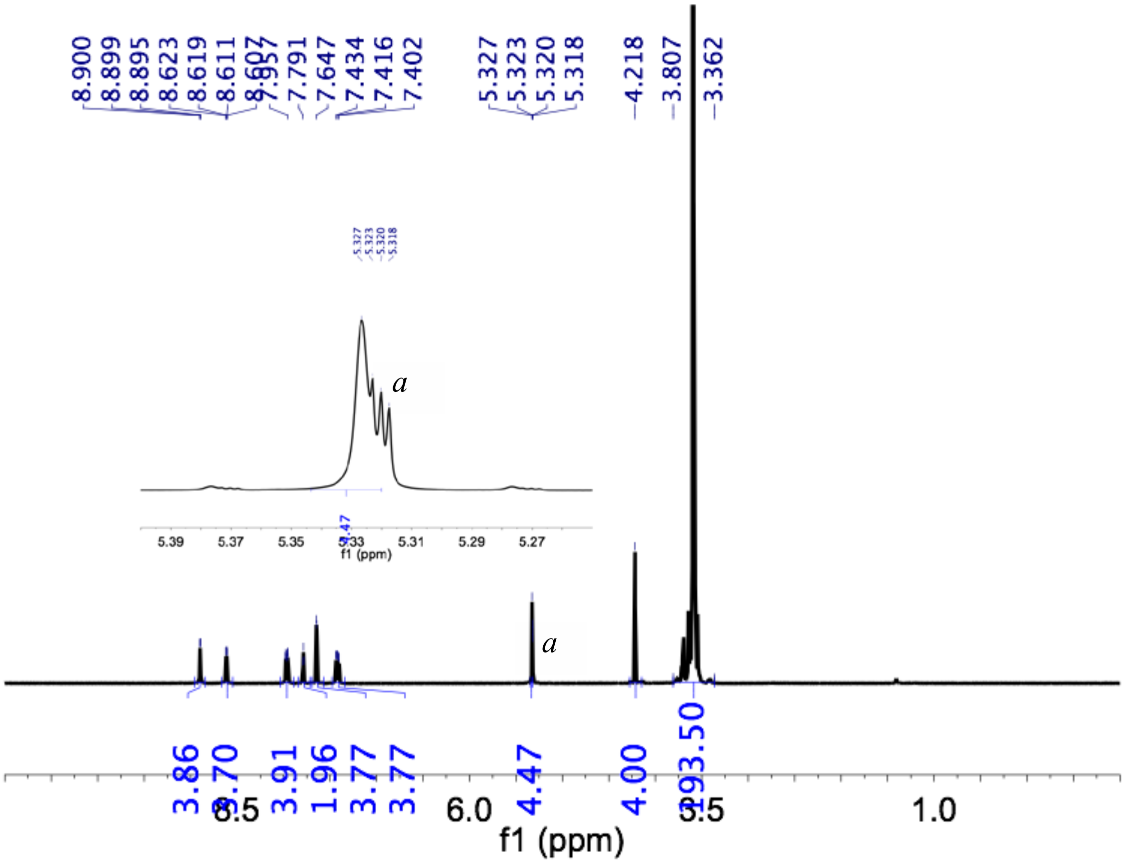

^{1}H NMR of **PL2** at 25 °C in CD_2Cl_2. [a] This resonance is due to residual $CHDCl_2$ (see inset).

© 2015 Macmillan Publishers Limited. All rights reserved

Supplementary Figure S20

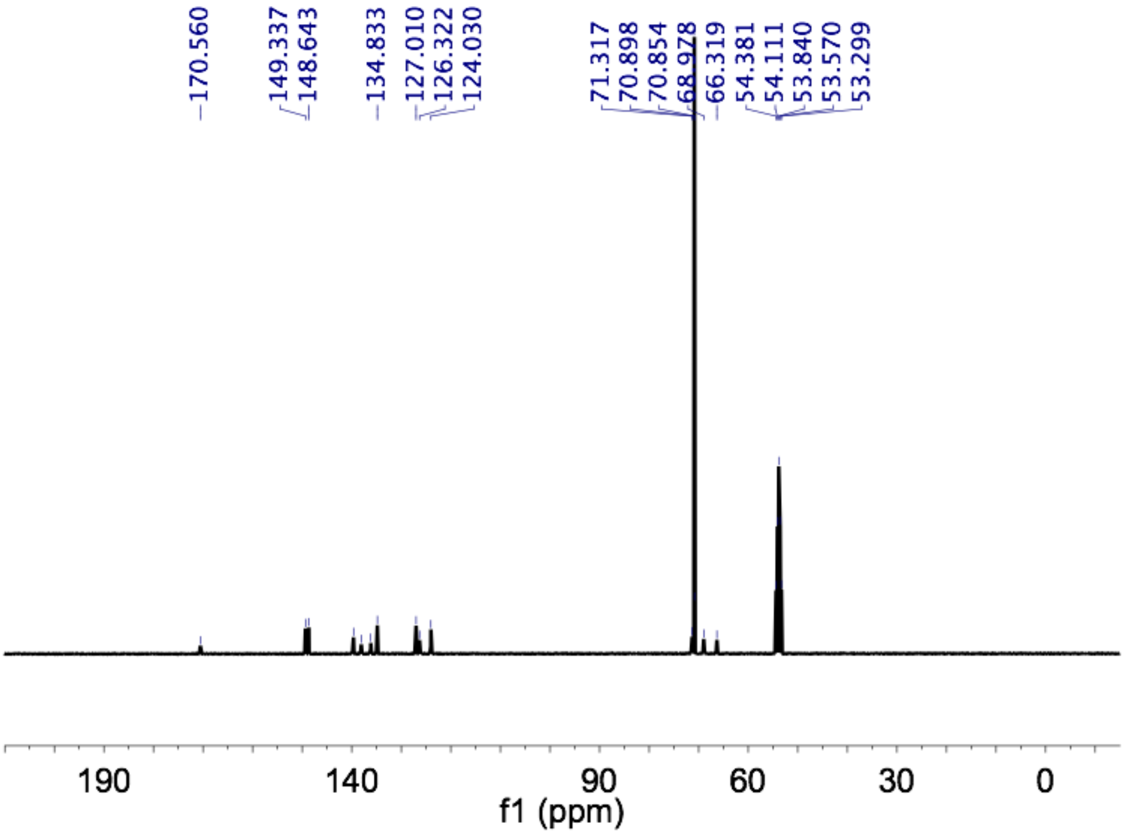

^{13}C NMR of **PL2** at 25 °C in CD_2Cl_2. [a] This resonance is due to CD_2Cl_2.

© 2015 Macmillan Publishers Limited. All rights reserved

Supplementary Figure S21

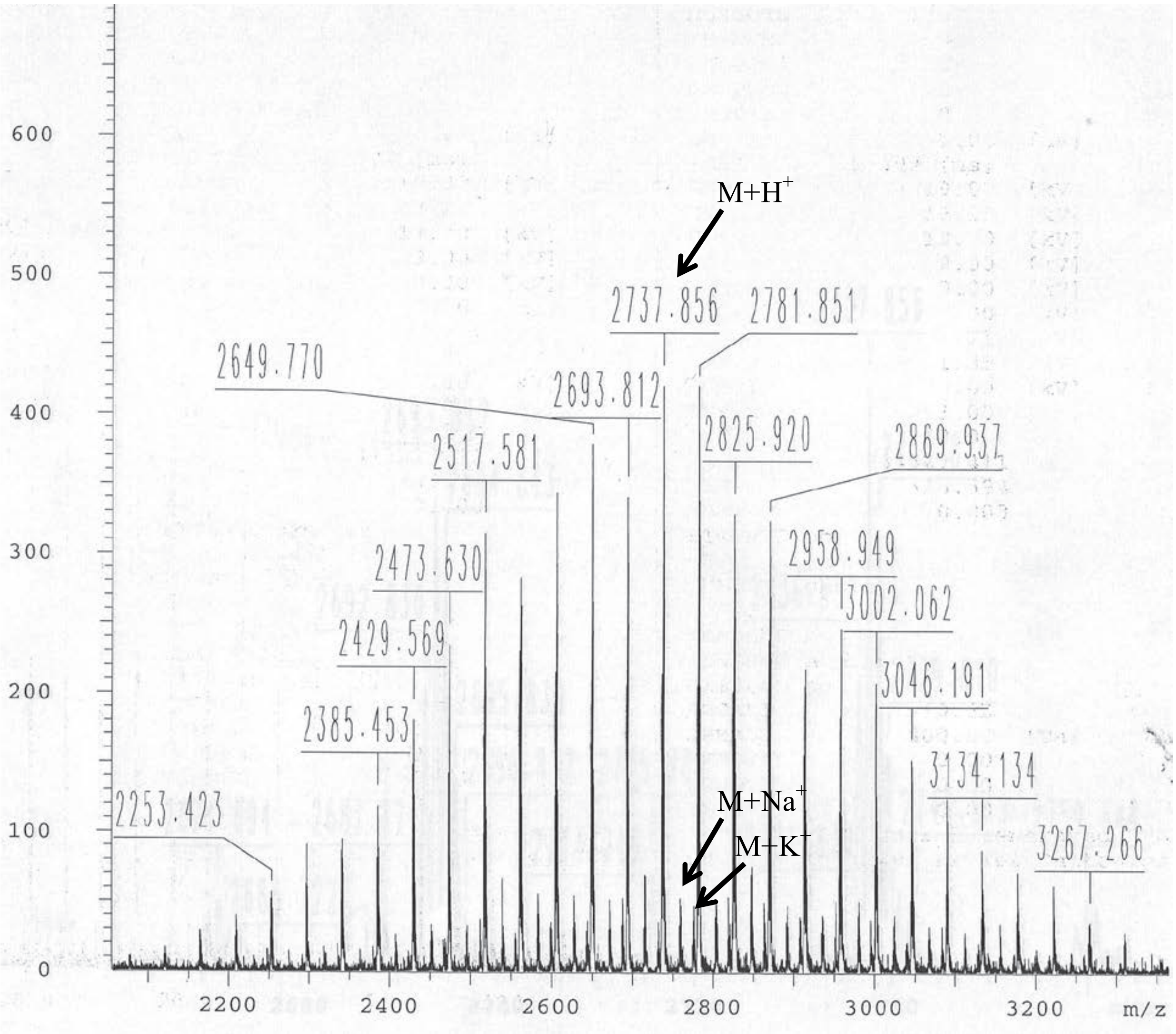

MALDI-TOF mass spectrum of **PL2**.

© 2015 Macmillan Publishers Limited. All rights reserved

Supplementary Figure S22

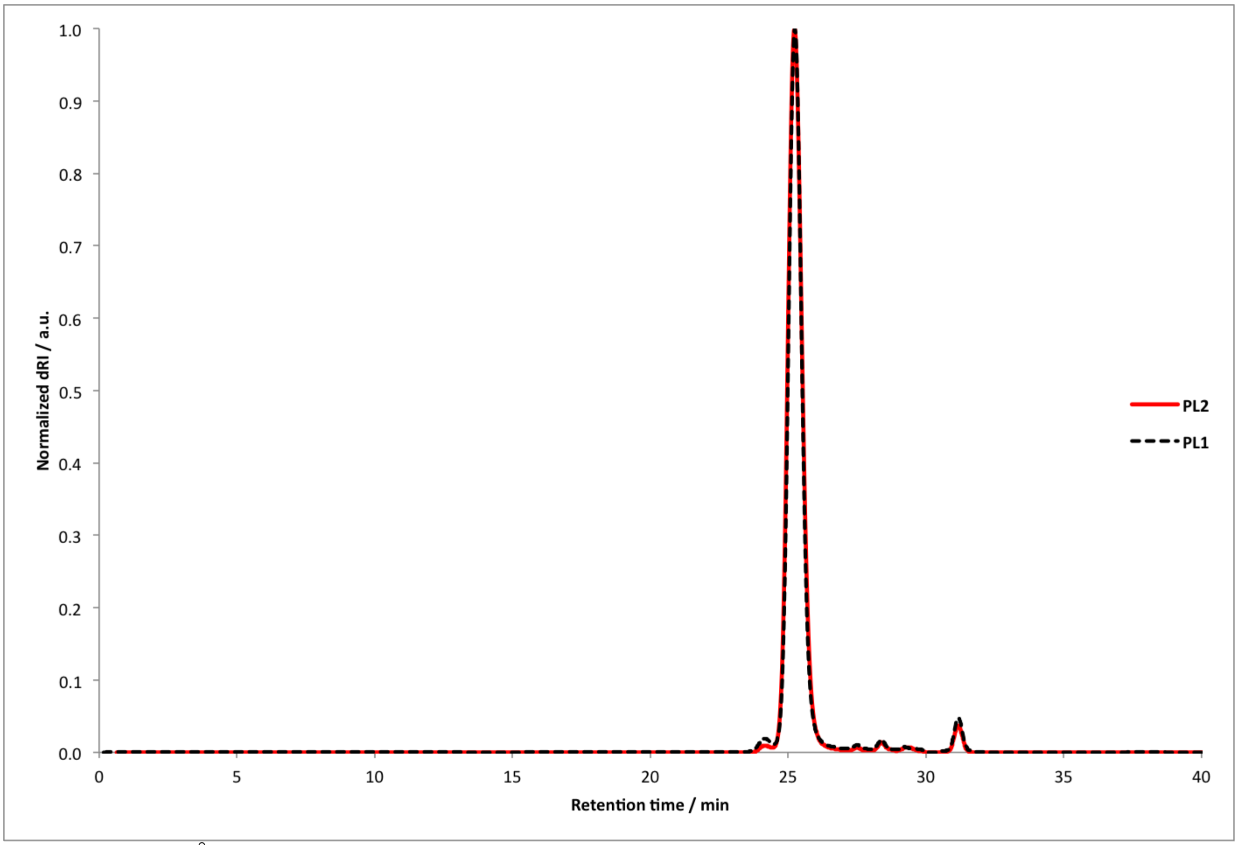

GPC (THF, 25 °C, *vide supra*) analysis of **PL1** and **PL2**. .

© 2015 Macmillan Publishers Limited. All rights reserved

Supplementary Figure S23

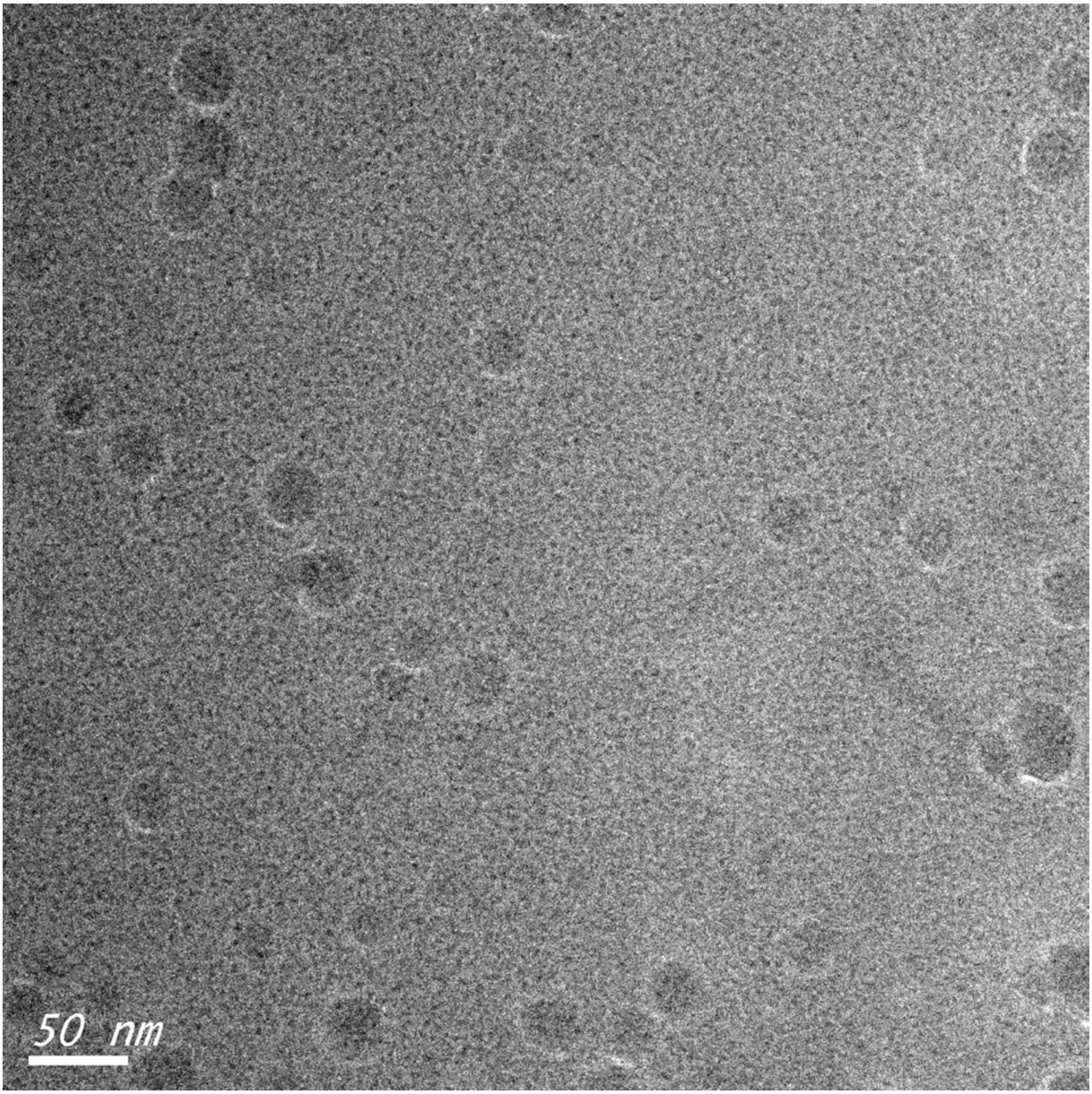

Cryo-TEM micrograph of the solutions of hyperbranched polymer networks, which consist of cages (and clusters of cages) prepared from **PL1** and $Pd(NO_3)_2$•$2H_2O$ at a sufficiently low concentration ([**PL1**] = 4.6 mM) to avoid gelation, dialyzed against Milli-Q® water (*vide supra*), The spherical features with a diameter of ~30-40 nm correspond to these clusters of cages.

© 2015 Macmillan Publishers Limited. All rights reserved

Supplementary Figure S24

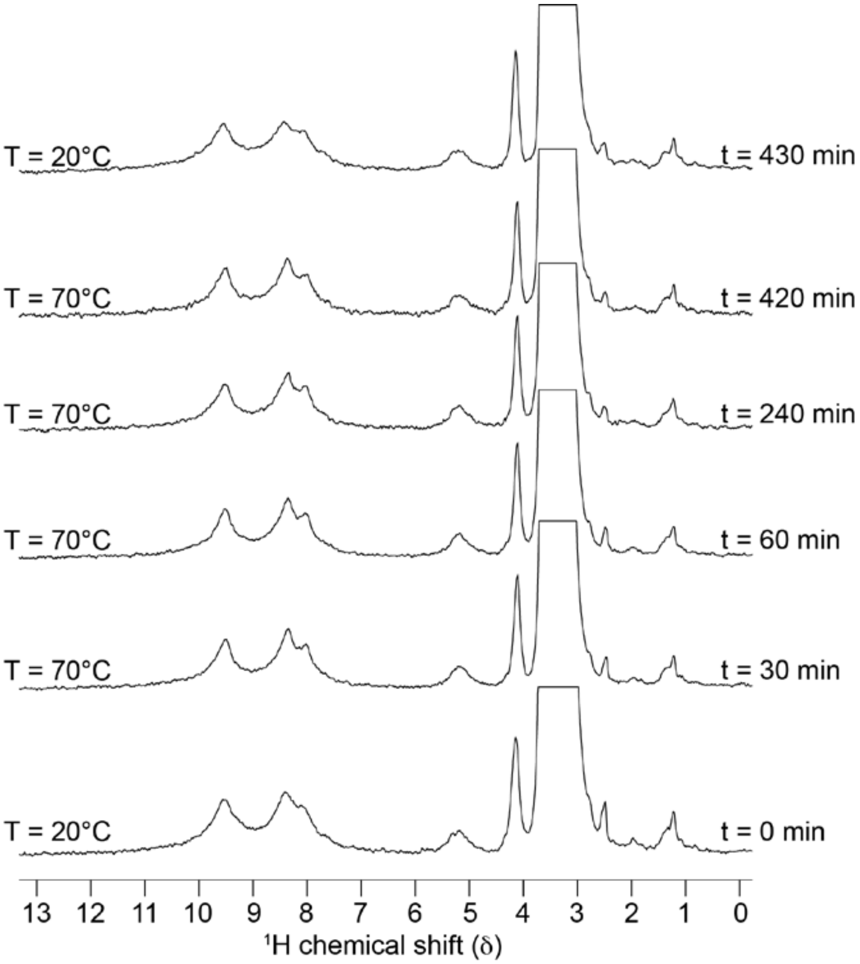

VT ^{1}H MAS NMR spectroscopy (500 MHz, DMSO-d^{6}, $\omega_{r/2}\pi$ = 10 kHz) of the **gel-1** ([**PL1**] = 24 mM) before, during, and after annealing.

© 2015 Macmillan Publishers Limited. All rights reserved

Supplementary Figure S25

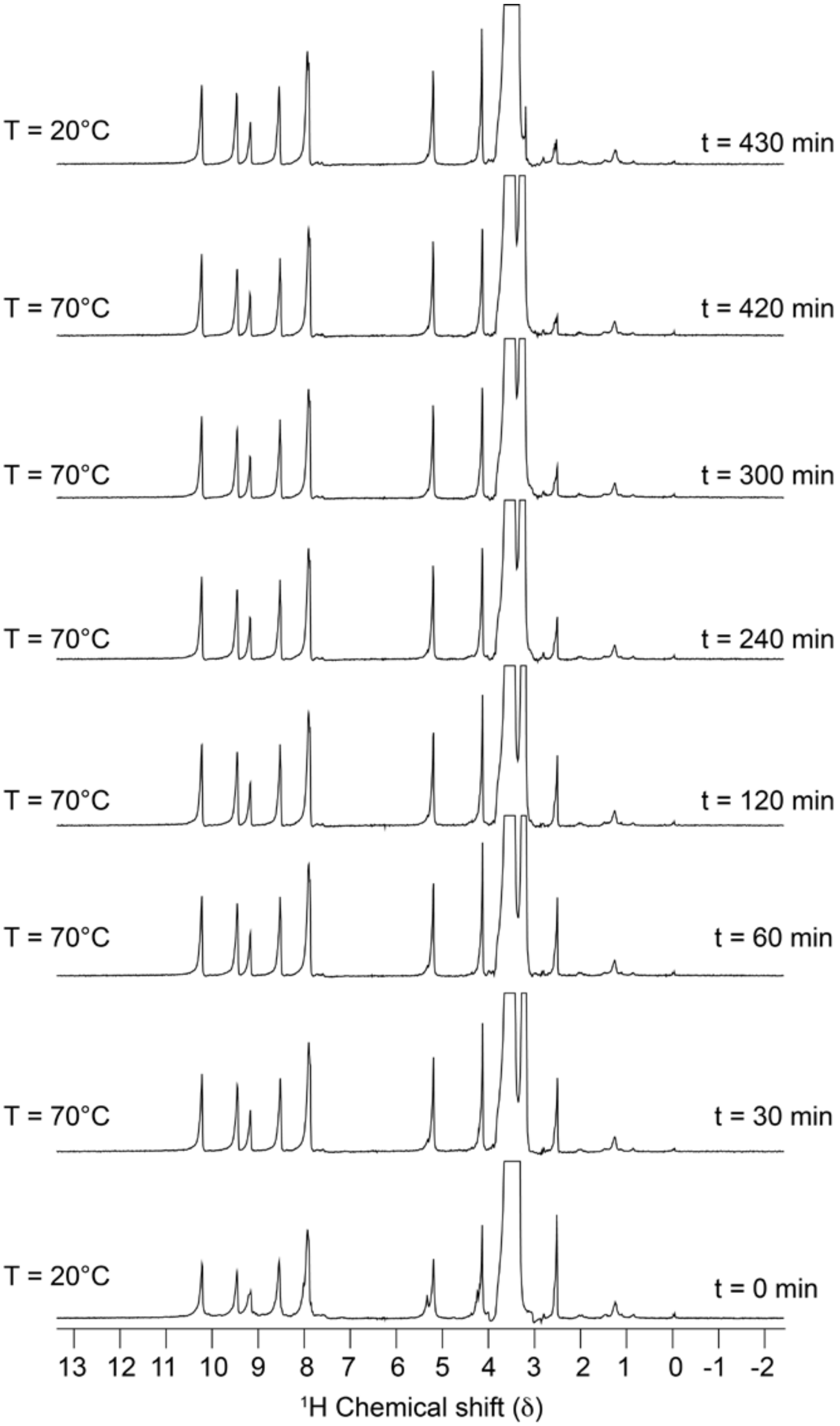

VT ^{1}H MAS NMR spectroscopy (500 MHz, DMSO-d^6, $\omega_{r/2}\pi$ = 10 kHz) of **gel-2** ([**PL2**] = 24 mM) before, during, and after annealing.

© 2015 Macmillan Publishers Limited. All rights reserved

Supplementary Figure S26

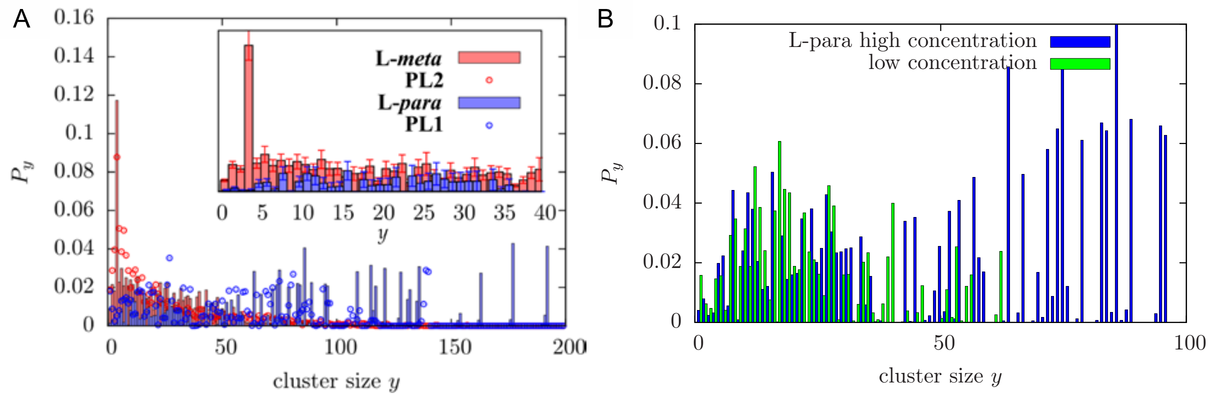

A. Distribution of cluster sizes for systems with **L-*para*** and **L-*meta*** ligands are plotted with blue or red bars, respectively. The data representing polyMOC networks derived from **PL1** and **PL2** are plotted with points using the same colors. B. The probability distribution for observing a cluster of size y in a low concentration simulation (system sidelength = 30 nm) is plotted with green boxes. For comparison, the similar distribution for high concentrations (system sidelength = 18.7 nm) is plotted in blue boxes, however scaled so that the normalization is equivalent on the increment of $0 \leq y \leq 50$.

© 2015 Macmillan Publishers Limited. All rights reserved

Supplementary Figure S27

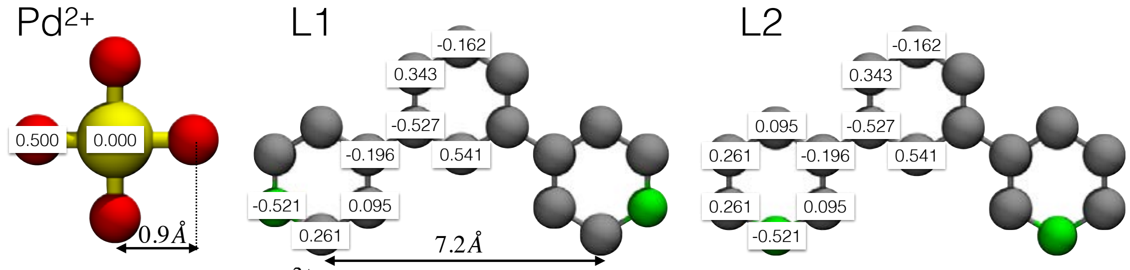

A rendering of the Pd^{2+} complex, the *para*-substituted bis-pyridine ligand (**L-*para***), and the *meta*-substituted bis-pyridine ligand (**L-*meta***). The atoms are labeled with their respective partial charges, given in units of electronic charge.

© 2015 Macmillan Publishers Limited. All rights reserved

Supplementary Figure S28

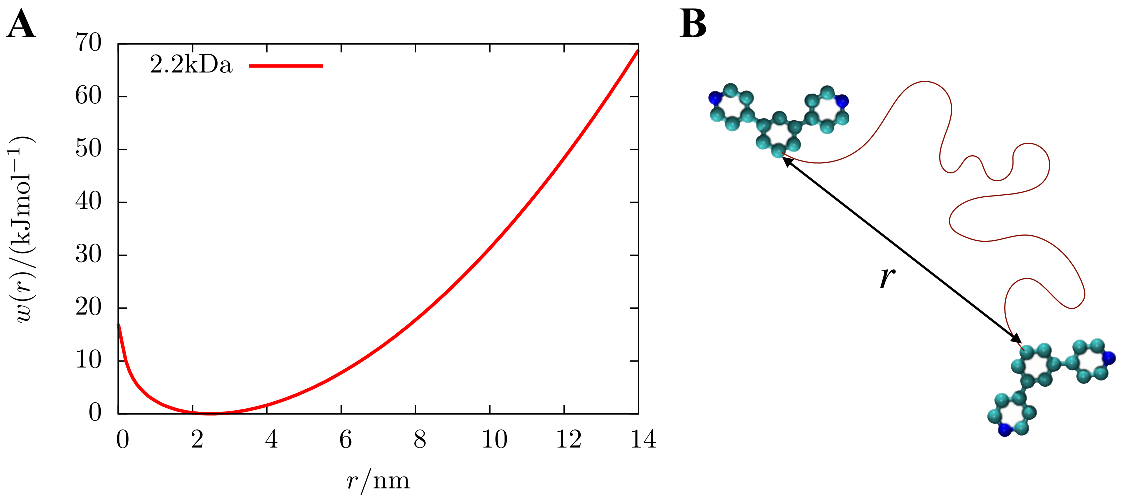

A. The ligand-ligand pair potential used for simulating macromers. **B.** The pair potential acts on the central carbon atoms of two specific ligands.

© 2015 Macmillan Publishers Limited. All rights reserved

Supplementary Figure S29

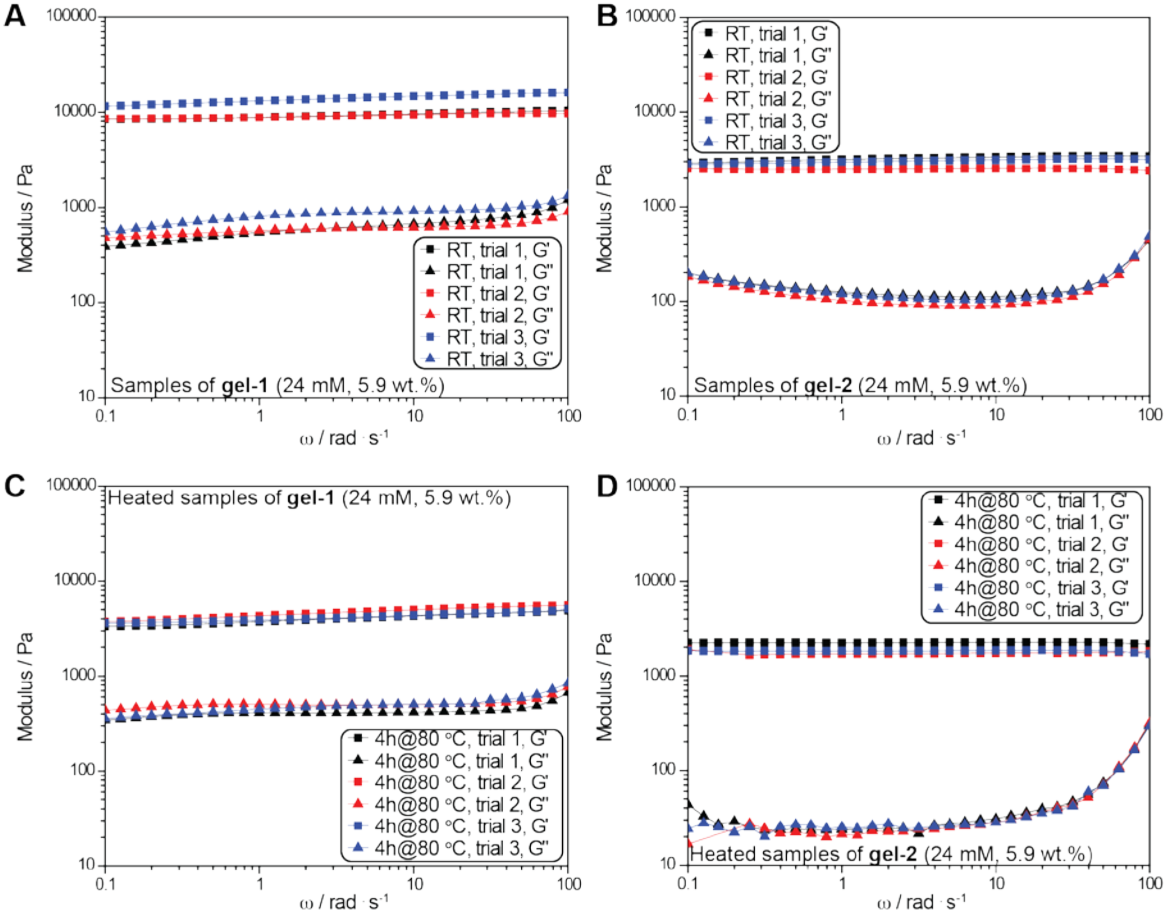

Frequency sweeps in oscillatory rheology at 1.0 % strain amplitude for **gel-1** ([**PL1**] = 24 mM) before (**A**) and after annealing for 4 h at 80 °C (**B**), and the same frequency sweep measurements for **gel-2** ([**PL2**] = 24 mM) before (**C**) and after annealing for 4 h at 80 °C (**D**).

© 2015 Macmillan Publishers Limited. All rights reserved

Supplementary Figure S30

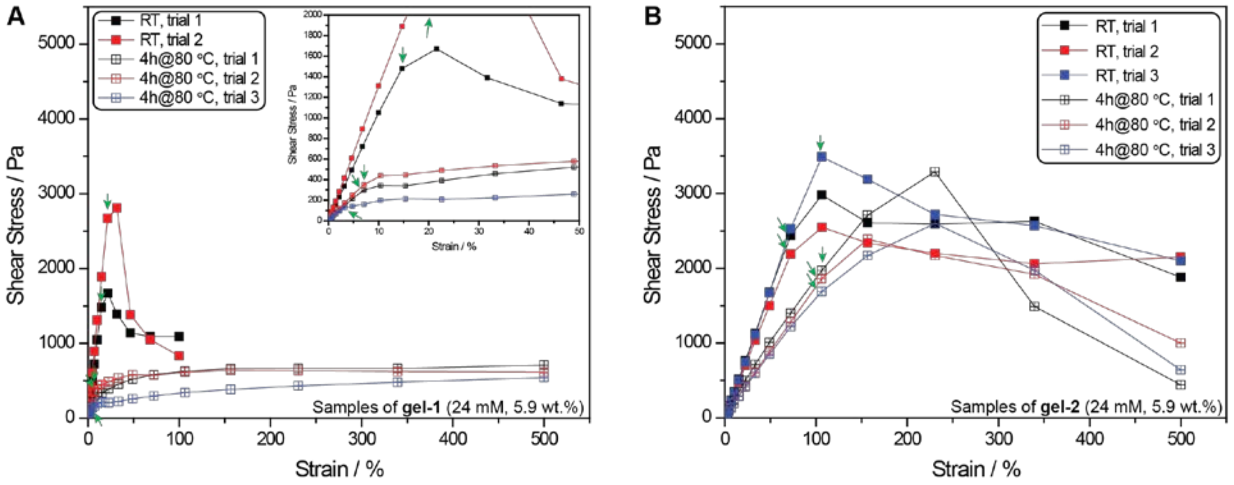

Stress *vs.* strain plots before and after thermal annealing for **gel-1** ([**PL1**] = 24 mM, **A**) and **gel-2** ([**PL2**] = 24 mM, **B**). Green arrows indicate points used for the determination of the yield stresses and strains.

© 2015 Macmillan Publishers Limited. All rights reserved

Supplementary Figure S31

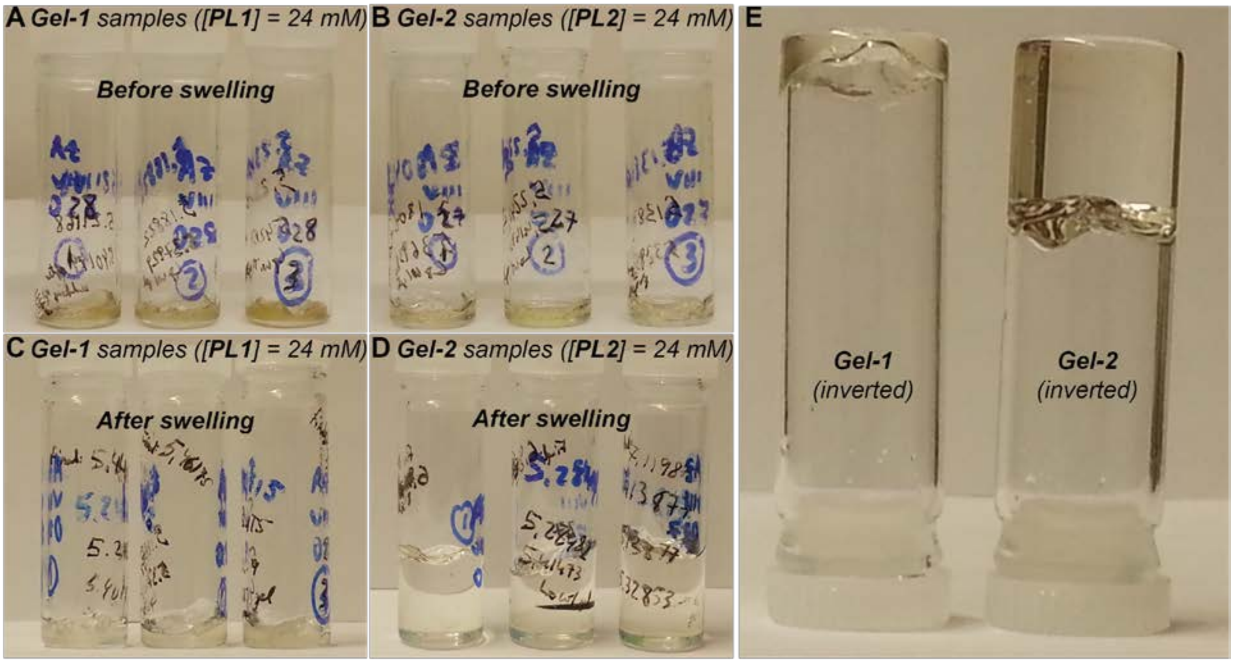

A. Three samples of **gel-1** ([**PL1**] = 24 mM) in 1-dram vials as prepared after annealing for 4 hours at 80 °C. **B.** Three samples of **gel-2** ([**PL2**] = 24 mM) in 1-dram vials as prepared after annealing for 4 hours at 80 °C. **C.** PolyMOC gels shown in panel **A** after swelling with 3.8 mL DMSO during the course of 5 days and then removing the excess DMSO. **D.** Gels shown in panel **B** after swelling with 3.8 mL DMSO during the course of 5 days and then removing the excess DMSO. **E.** Inversion test on the swollen polyMOCs confirms that they are still gels.

© 2015 Macmillan Publishers Limited. All rights reserved

Supplementary Figure S32

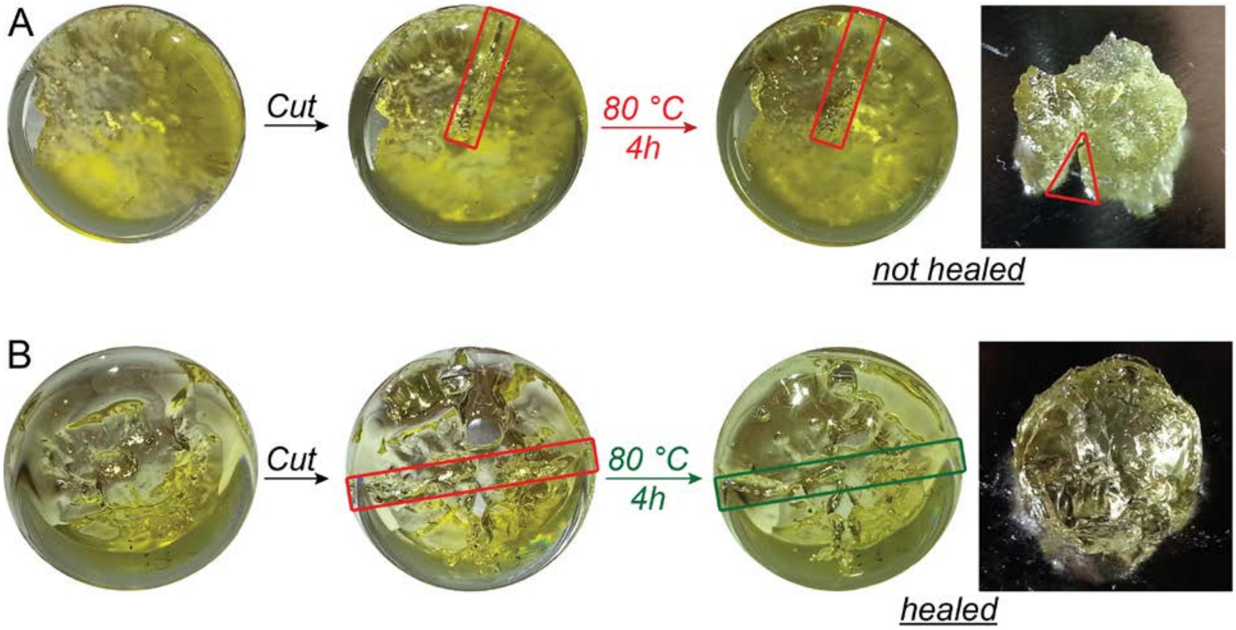

A. **Gel-1** in DMSO-d^6 ([**PL1**] = 24 mM) as prepared (left), after cutting into two pieces with a spatula blade (center left, cut region marked with a red box), and after annealing for 4 h at 80 °C (center right), respectively. These photographs were taken from the bottom of a 1-dram vial, which contained the gel. The unhealed cut is easily visualized by separating the two parts of the gel around the cut (highlighted by the red triangle in the photo on the right). B. **Gel-2** in DMSO-d^6 ([**PL2**] = 24 mM) as prepared (left), after cutting into two pieces with a spatula blade (center left, cut region marked with a red box), and after annealing for 4 h at 80 °C (center right), respectively. These photographs were taken from the bottom of a 1-dram vial, which contained the gel. The damage was visibly healed in the region highlighted by the green box; the photo on the right depicts the monolith nature of the healed gel. No solvent was added or pressure applied to facilitate healing.

© 2015 Macmillan Publishers Limited. All rights reserved

Supplementary Figure S33

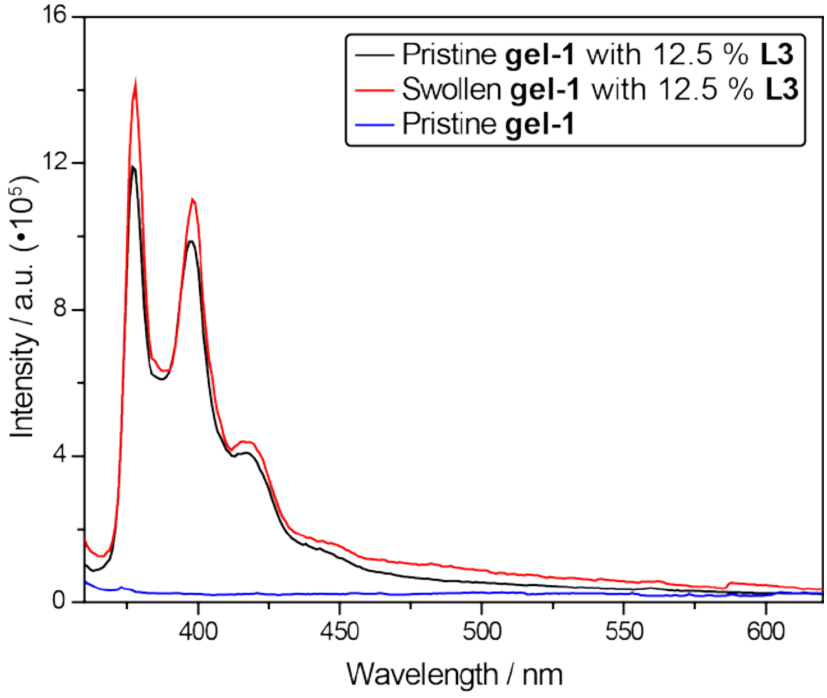

Fluorescence spectra of pristine **gel-1** gel (5.9 wt. %, blue trace), and the same gels with 12.5% **PL1** replaced with **L3** before (black) and after (red trace) swelling with DMSO (~66-fold excess) for 2 d; the "after swelling" fluorescence spectrum (red) has been scaled by the ratio of gel mass before and after swelling (264.5 mg/126.7 mg), since approximately identical amounts of gels were used for the fluorescence measurements (*vide supra*).

© 2015 Macmillan Publishers Limited. All rights reserved

Supplementary Figure S34

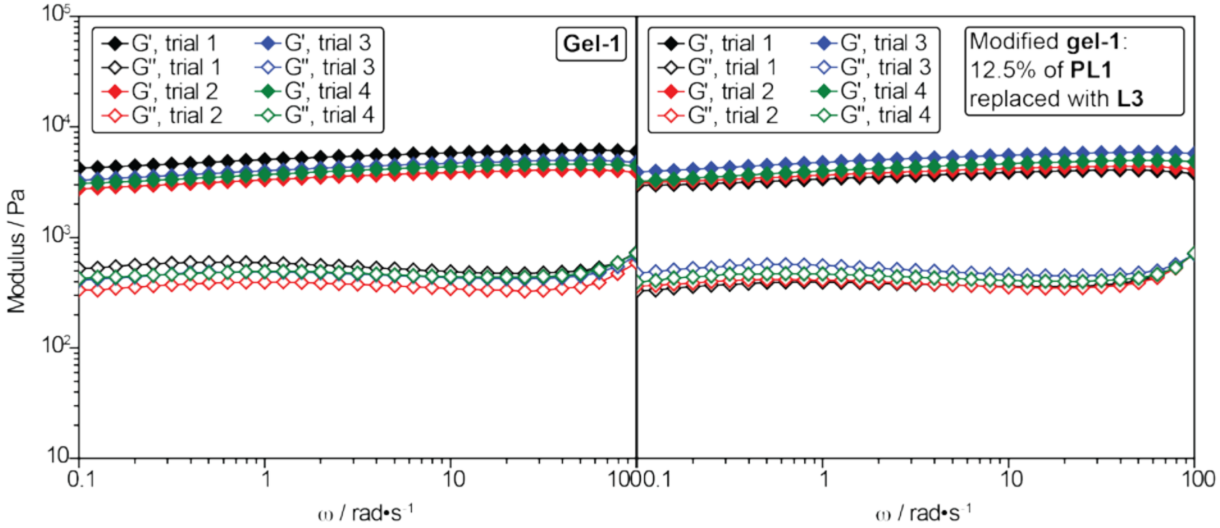

Frequency sweeps in oscillatory rheology at 1.0 % strain amplitude for four different samples of **gel-1** ([**PL1**] = 24 mM) after annealing for 4 h at 80 °C (left), and the same frequency sweep measurements for four different samples **gel-1** with 12.5 % **PL1** replaced with **L3** after annealing for 4 h at 80 °C (right). The *G′* for **gel-1** gels is 4.7 ± 0.8 kPa, and the *G′* of the **L3**-modified **gel-1** gels is 4.6 ± 0.7 kPa. Note that a new batch of **PL1** was used for these measurements.

© 2015 Macmillan Publishers Limited. All rights reserved

Supplementary Table S1

Rheometry characterization of the polyMOC gels via frequency sweeps

Macromer used for gel	G' (100 rad/s), initial / Pa	G''(100 rad/s), initial / Pa	G' (100 rad/s), annealed /Pa	G''(100 rad/s), annealed / Pa
**PL1**	10377	1202.1	4869.4	664.34
**PL1**	9677.4	895.03	5531	768.5
**PL1**	16011	1332.7	5090.1	842.28
**PL2**	3392	440	2160.6	303.51
**PL2**	2374.1	450.28	1805.5	317.41
**PL2**	3150.3	486.15	1703.6	292.29

© 2015 Macmillan Publishers Limited. All rights reserved

Supplementary Table S2

Rheometry characterization of the polyMOC gels via strain sweeps

Macromer used for gel	Yield stress, initial / Pa	Yield strain, initial / %	Yield stress, annealed / Pa	Yield strain, annealed / %
**PL1**	1480	14.7	298	7.07
**PL1**	2670	21.5	351	7.07
**PL1**			142	4.81
**PL2**	2980	106	1970	106
**PL2**	2190	72.2	1860	106
**PL2**	2530	72.2	1690	106

© 2015 Macmillan Publishers Limited. All rights reserved

Supplementary Table S3

Swelling ratio quantification for the polyMOC gels derived from **PL1** and **PL2**

Macromer used to make the gel	Dry mass (g)	Mass of swollen gel (g)	Swelling ratio
**PL1**	0.01113	0.23241	20.9
**PL1**	0.01113	0.27353	24.6
**PL1**	0.01113	0.25938	23.3
**PL2**	0.01113	1.71175	154
**PL2**	0.01113	1.65467	149
**PL2**	0.01113	1.85764	167

© 2015 Macmillan Publishers Limited. All rights reserved

Supplementary Table S4

The effect of % macromer replaced on *G'*, *G''* and *f* of polyMOC gels (averages of 3-4 trials)

% Macromer replaced	G'(10 rad/s) of **gel-1**/ Pa	G''(10 rad/s) of **gel-1** / Pa	G'(10 rad/s) of **gel-2** / Pa	G''(10 rad/s) of **gel-2** / Pa	*f* of **gel-1**	*f* of **gel-2**
0	5989.8	554.1	1780.1	49.0	4.45	2.12
6.25	5487.0	465.5			4.24	
12.5	5726.2	374.4	561.4	23.1	4.33	2.04
18.75	2974.5	245.6			3.20	
25	2952.8	209.0	111.8	11.4	3.19	2.01
33.333	1909.5	151.6			2.77	
41.67	1355.8	92.2			2.54	
50	740.59	68.4			2.29	

© 2015 Macmillan Publishers Limited. All rights reserved
